# GluN2B suppression restores phenylalanine-induced neuroplasticity and cognition impairments in a mouse model of phenylketonuria

**DOI:** 10.1172/JCI184299

**Published:** 2025-05-08

**Authors:** Woo Seok Song, Young Sook Kim, Young-Soo Bae, Sang Ho Yoon, Jae Min Lim, Myoung-Hwan Kim

**Affiliations:** 1 Department of Physiology and Biomedical Sciences, Seoul National University College of Medicine, Seoul, South Korea.; 2 Neuroscience Research Institute, Seoul National University Medical Research Center, Seoul, South Korea.; 3 Seoul National University Bundang Hospital, Seongnam, Gyeonggi, South Korea.

**Keywords:** Metabolism, Neuroscience, Intellectual disability, Synapses

## Abstract

Phenylketonuria (PKU), an inborn error of phenylalanine (Phe) metabolism, is a common cause of intellectual disability. However, the mechanisms by which elevated Phe levels cause cognitive impairment remain unclear. Here, we show that submillimolar Phe perturbs synaptic plasticity through the hyperactivation of GluN2B-containing NMDARs. *Pah^Enu2^* PKU model mice exhibited submillimolar and supramillimolar concentrations of Phe in the cerebrospinal fluid (CSF) and serum, respectively. l-Phe produced concentration-dependent bidirectional effects on NMDA-induced currents, without affecting synaptic NMDA receptors (NMDARs) in hippocampal CA1 neurons. l-Phe-induced hyperactivation of extrasynaptic GluN2B resulted in activity-dependent downregulation of AMPA receptors during burst or sustained synaptic activity. Administration of l-Phe in mice decreased neural activity and impaired memory, which were blocked by pretreatment with GluN2B inhibitors. Furthermore, pharmacological and virus-mediated suppression of GluN2B reversed the impaired learning in *Pah^Enu2^* mice. Collectively, these results suggest the concentration of Phe in the CSF of patients with PKU perturbs extrasynaptic NMDARs and synaptic plasticity and that suppression of GluN2B may have the potential to improve cognitive function in patients with PKU.

## Abstract 2

This study reveals the synaptic mechanisms underlying cognitive impairment in phenylketonuria that brain concentration of phenylalanine hyperactivates GluN2B-containing extrasynaptic NMDARs and perturbs synaptic plasticity.

## Graphical abstract

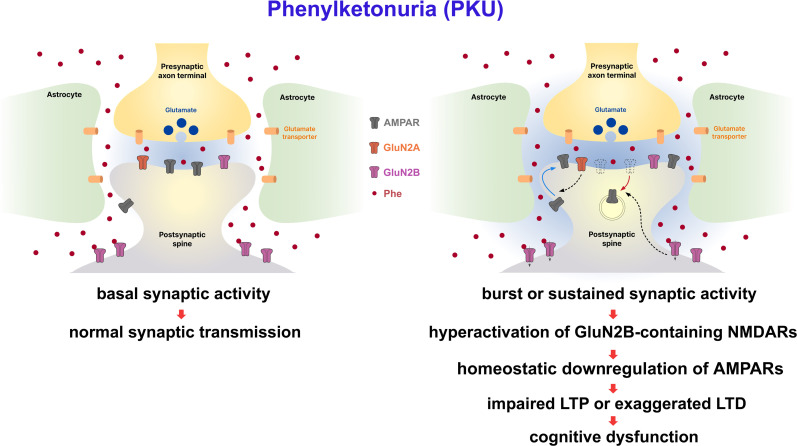

## Introduction

Phenylketonuria (PKU), the most common inborn error of metabolism, affects approximately 450,000 individuals worldwide (1). Deficiencies in the activity of phenylalanine hydroxylase (PAH), which catalyzes the conversion of phenylalanine (Phe) to tyrosine (Tyr) in the liver, lead to Phe accumulation in the blood and tissues (2, 3). Phe in the blood is transported across the blood-brain barrier (BBB) and results in an elevated Phe concentration in the brain. PKU causes severe intellectual disability; the intelligence quotient (IQ) score of untreated patients with PKU is less than 50 (4). Treatment with restricted Phe intake (low-Phe diet) starting at an early age prevents severe intellectual disabilities. However, abnormal cognitive outcomes have been consistently observed in both continuously and early treated patients with PKU (_ETP_PKU) (5–9).

Multiple hypotheses, including white matter abnormalities (WMAs), insufficient Tyr and tryptophan levels, reduced catecholamine and serotonin levels, and elevated Phe levels, have been proposed as neural mechanisms for cognitive impairment in patients with PKU. Despite the high rate of WMAs, probably due to reduced cerebral protein synthesis (10), in both untreated patients and _ETP_PKU (11–14), clinical studies have found no significant association between the extent of WMAs and cognitive outcomes (12–16). In addition, blood Tyr concentrations do not correlate well with cognitive outcomes, and Tyr or l-dihydroxyphenylalanine supplementation has no cognitive benefits (17). Deficiencies in tryptophan or serotonin are thought to be associated with psychosocial dysfunction rather than cognitive impairments (18). Meanwhile, a clear correlation between Phe levels and cognitive impairment has been consistently observed in _ETP_PKU (5, 6, 15, 19, 20). However, the mechanisms that link elevated Phe levels to cognitive impairment remain unclear.

Previous studies have demonstrated that l-Phe, at concentrations observed in or higher than that in PKU serum, reduces currents mediated by NMDA receptors (NMDARs) and AMPA receptors (AMPARs) in cultured hippocampal and cortical neurons (21–23). However, accumulating evidence from clinical and preclinical studies has revealed that the Phe concentration in the brain (Phe_brain_) or cerebrospinal fluid (Phe_CSF_) is substantially lower than that in the blood (Phe_blood_) in patients with PKU and in *Pah^Enu2^* PKU mouse models (24–29). The mean blood-brain ratios of Phe in _ETP_PKU were 4.12 (25) and 4.0 (28) whereas the mean Phe_brain_ has been measured as approximately 250 μmol/L (25, 26, 28). Even in untreated children with classical PKU, the mean Phe_CSF_ was 399 μmol/L (24). Notably, oral Phe loading in _ETP_PKU increased the Phe_brain_ from 250 to 400 μmol/L and concomitantly shifted the dominant peak of the EEG background activity to the lower-frequency spectrum (26). These observations indicate that submillimolar Phe levels affect neural circuits and brain activity in individuals with PKU. However, a central, unresolved question remains: How do submillimolar Phe concentrations cause cognitive impairment in patients with PKU?

Although synaptic dysfunction is commonly associated with cognitive impairment, the impact of elevated Phe levels on activity-dependent modification of synaptic efficacy remains unclear. Moreover, the effects of Phe_CSF_, at concentrations observed in patients with PKU, on AMPAR- and NMDAR-mediated synaptic transmission at central synapses may differ from those of supramillimolar Phe.

To understand the neurophysiological mechanisms underlying cognitive impairments in PKU, we investigated the effects of submillimolar Phe on synaptic transmission and plasticity in CA1 neurons in acute hippocampal sections. We show that Phe in the PKU CSF hyperactivates GluN2B and perturbs synaptic plasticity via activity-dependent downregulation of AMPARs.

## Results

### Effects of l-Phe on NMDAR currents.

**Figure 1:**
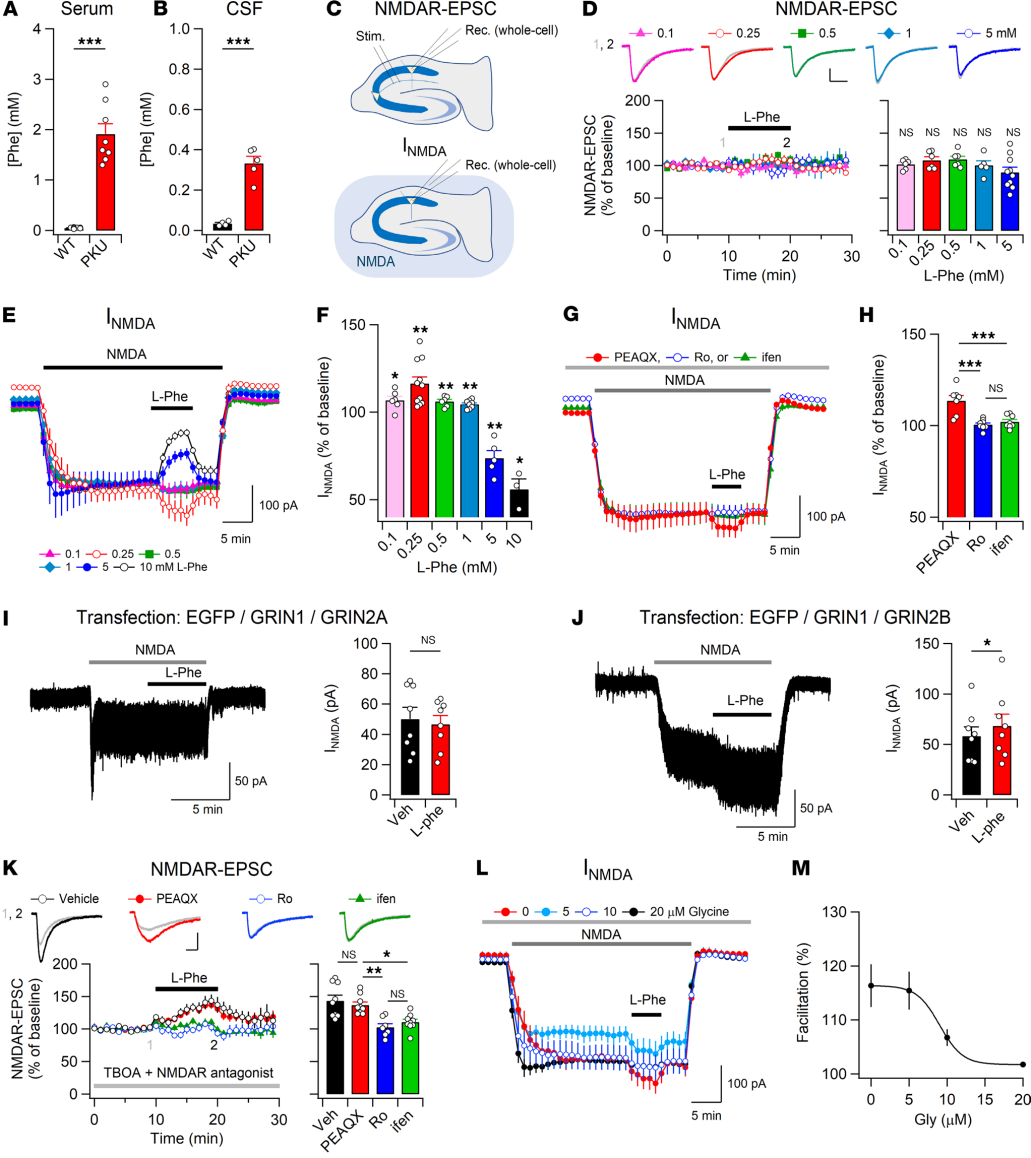
Submillimolar l-Phe increases GluN2B-NMDAR activity. (**A** and **B**) Increased Phe concentration in the serum (**A**) and CSF (**B**) of adult *Pah^Enu2^* mouse. (**C**) Experimental design for NMDAR-EPSC (top) and I_NMDA_ (bottom) recordings. Stim., stimulating electrode; Rec., recording electrode. (**D**) Representative traces of NMDAR-EPSCs (top) obtained at the indicated time points (1, 2), and the time course of the peak amplitudes (bottom left) of NMDAR-EPSCs measured at −40 mV in CA1 neurons. Bottom right: l-Phe had no effect on the peak amplitudes of NMDAR-EPSCs. (**E**) In the presence of NBQX and picrotoxin, I_NMDA_ was induced by bath application of NMDA (5−10 μM). Different concentrations of l-Phe were perfused with NMDA for 5 minutes. (**F**) l-Phe exhibits concentration-dependent bidirectional effects on I_NMDA_. (**G**) I_NMDA_ was induced by 3−12 μM NMDA in the presence of GluN2A or GluN2B blockers. (**H**) l-Phe-induced facilitation of I_NMDA_ was blocked by Ro (2 μM) or ifen (6 μM) but not by PEAQX (0.5 μM). (**I** and **J**) A sample trace (left) and summary (right) of I_NMDA_ measured before and during l-Phe perfusion in HEK293 cells expressing GluN2A (**I**) or GluN2B (**J**). (**K**) Representative traces (top) and the peak amplitudes (bottom left) of NMDAR-EPSCs measured in the presence of TBOA (10 μM). l-Phe induced facilitation of NMDAR-EPSCs in each condition (bottom right). (**L**) Addition of 5, 10, and 20 μM glycine attenuated l-Phe-induced I_NMDA_ facilitation. I_NMDA_ was induced by 5 μM NMDA. (**M**) The concentration relationship between l-Phe-induced facilitation of I_NMDA_ and added glycine (Gly) concentration. A Student’s *t* test (**A**, **B**, **D**, **F**, **I**, and **J**) or a 1-way ANOVA with a post hoc Tukey’s test (**H** and **K**) was used for statistical analysis. *P < 0.05, **P < 0.01, ***P < 0.001, and NS, *P* ≥ 0.05. Scale bars: 50 ms and 50 pA (**D** and **K**) Veh, vehicle.

We first investigated Phe concentrations in the serum and CSF of PKU (*Pah^Enu2^*) mice. *Pah^Enu2^* mice had significantly higher levels of Phe in both the serum and CSF compared with WT mice (Figure 1, A and B). However, consistent with previous reports (29), Phe_CSF_ was significantly lower than Phe concentration in serum (Phe_Serum_) in *Pah^Enu2^* mice (*t*_(11)_ = 5.88, *P* = 0.000106 by Student’s *t* test).

The influence of CSF Phe levels in PKU on NMDAR-mediated synaptic transmission remains unknown. Therefore, we examined the effects of various concentrations of l-Phe on NMDAR-mediated excitatory postsynaptic currents (NMDAR-EPSCs) at the Schaffer collateral (SC)-CA1 synapse, as well as on the current induced by NMDA perfusion (I_NMDA_) in CA1 pyramidal neurons, using hippocampal sections from adult (6- to 9-week-old) mice (Figure 1C). Unexpectedly, the peak amplitudes of NMDAR-EPSCs at this synapse were not affected by any of the tested concentrations (0.1–5 mM) of l-Phe (Figure 1D). However, in contrast to synaptic transmission, l-Phe had a concentration-dependent bidirectional effect on the I_NMDA_ (Figure 1, E and F): l-Phe concentrations lower than 1 mM significantly enhanced the I_NMDA_, with the maximum effect observed at 250 μM, which is fairly close to the mean CSF concentration of l-Phe observed in *Pah^Enu2^* mice (Figure 1B) and patients with PKU (24–28). In contrast, higher concentrations (5 and 10 mM) of l-Phe, similar to previous studies (21, 22), significantly reduced I_NMDA_. Because GluN2A and GluN2B are the main types of NMDAR subunit in the forebrain (30), we examined the action of 250 μM l-Phe on each type of NMDAR. The enhancement in I_NMDA_ caused by l-Phe was sensitive to antagonists selective for GluN2B but not for GluN2A-containing NMDARs (Figure 1, G and H). Consistent with these observations, l-Phe increased I_NMDA_ induced by NMDA (30 μM) perfusion in HEK293 cells expressing human GluN1 and GluN2B receptors but not in HEK293 cells expressing human GluN1 and GluN2A receptors (Figure 1, I and J), indicating a direct and preferential action of l-Phe on GluN2B-containing NMDARs.

We wondered whether the absence of any effect of l-Phe on NMDAR-EPSCs stemmed from the subcellular location of NMDARs (31, 32). In the presence of the glutamate reuptake inhibitor dl-*threo*-β-benzyloxyaspartic acid (TBOA), which promotes glutamate spillover to extrasynaptic areas (33), l-Phe significantly enhanced NMDAR-EPSCs, indicating that l-Phe primarily influences the activity of extrasynaptic NMDARs at SC-CA1 synapses (Figure 1K). Similar to I_NMDA_, the effect of l-Phe on NMDAR-EPSCs was still observed under GluN2A inhibition but was blocked by the GluN2B inhibitors Ro25-6891 (Ro) and ifenprodil (ifen). The absence of an effect of l-Phe on NMDAR-EPSCs is unlikely to stem from the saturation of synaptic NMDARs (34). In the absence of TBOA, the addition of d-serine, but not glycine (32), to artificial CSF (ACSF) significantly increased the amplitude of NMDAR-EPSCs (Supplemental Figure 1; supplemental material available online with this article; https://doi.org/10.1172/JCI184299DS1).

Previous studies have reported that l-Phe competes for the glycine-binding site in NMDARs and that the attenuation of I_NMDA_ at high concentrations of l-Phe is dependent on the concentration of glycine (21, 22). Glycine exhibits approximately 10-fold higher affinity for GluN2B- than GluN2A-containing NMDARs (35). Because l-Phe-induced facilitation of I_NMDA_ and NMDAR-EPSCs under TBOA treatment was observed at ambient glycine levels in hippocampal slices, we tested whether the levels of glycine would affect the l-Phe-induced facilitation of I_NMDA_ in mouse hippocampal slices. The addition of glycine to ACSF decreased the magnitude of l-Phe-induced I_NMDA_ facilitation in a concentration-dependent manner (Figure 1, L and M). However, facilitation of I_NMDA_ was still observed at up to 10 μM glycine, which is the normal concentration in the human CSF (36). Considering that the CSF glycine concentration was markedly reduced in untreated infants with PKU but was within the normal range in children and adults with PKU (37, 38), Phe levels in the PKU CSF likely facilitate the neurotransmission and signaling of GluN2B-containing NMDARs.

### Normal NMDAR and AMPAR function and tonic GABAergic inhibition in Pah^Enu2^ mice.

**Figure 2:**
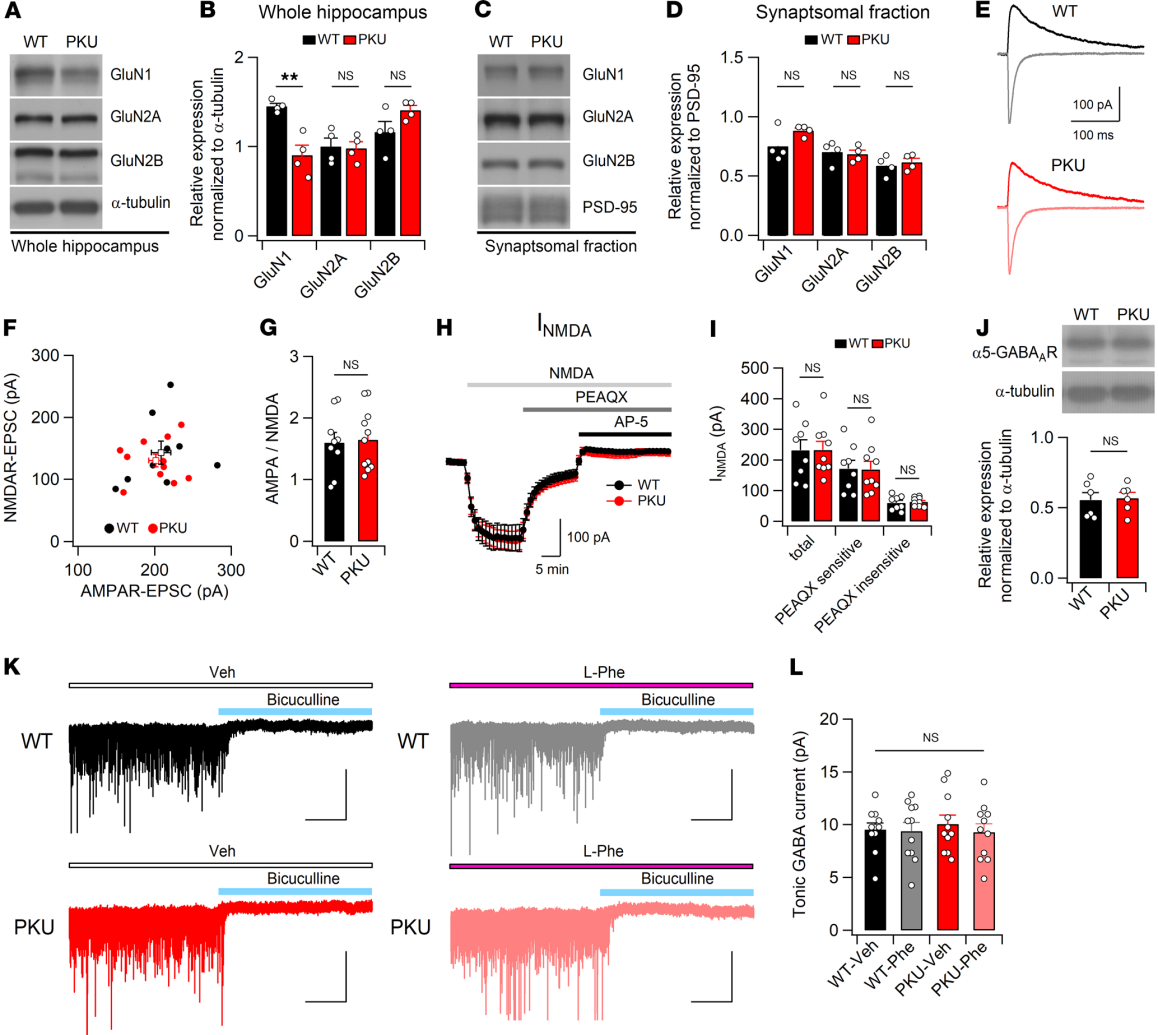
Normal NMDAR function and tonic GABAergic inhibition in the hippocampus of *Pah^Enu2^* mice. (**A**–**D**) Representative Western blots and expression levels of NMDAR subunits in the total (**A** and **B**) and synaptosomal fractions (**C** and **D**) of WT and *Pah^Enu2^* hippocampi. (**E**) Representative AMPAR- and NMDAR-EPSCs measured at −70 and +40 mV, respectively, in CA1 pyramidal neurons. (**F**) The peak amplitudes of NMDAR-EPSCs were plotted against AMPAR-EPSCs. (**G**) Normal AMPA to NMDA ratios in the CA1 pyramidal cells of *Pah^Enu2^* mice. (**H**) Bath application of NMDA (5 μM) induced similar magnitudes of inward current in CA1 pyramidal neurons of WT and *Pah^Enu2^* mice. I_NMDA_ was measured in the presence of NBQX and picrotoxin. PEAQX (0.5 μM) reduced I_NMDA_, and subsequent AP-5 (50 μM) perfusion blocked the remnant. (**I**) Total I_NMDA_ and PEAQX-sensitive and -insensitive components after sequential application of PEAQX and AP-5 in CA1 pyramidal cells. (**J**) Hippocampal expression levels (bottom) of α5-GABA_A_R were determined by Western blotting (top). (**K**) Representative traces of tonic currents measured from WT and *Pah^Enu2^* hippocampal CA1 neurons in the presence and absence of Phe. Vehicle (Veh) or Phe (250 μM) were perfused throughout the recordings. Scale bar: 30 seconds and 100 pA. (**L**) Magnitudes of bicuculline (40 μM)-sensitive tonic current in each condition are summarized. Statistical analysis was performed using Student’s *t* test (**B**, **D**, **G**, **I**, and **J**) and 2-way ANOVA (**L**). **P < 0.01 and NS, *P* ≥ 0.05.

Cognitive impairments in PKU are attributable to altered synaptic function caused by long-term exposure to elevated Phe and/or Phe in the CSF. Therefore, we examined whether the expression levels of the NMDAR subunits were altered in the brains of PKU mice (*Pah^Enu2^*). In contrast to the changes in glutamate receptor expression observed in the BTBR strain (22, 39), adult PKU mice with a C57BL/6N background exhibited reduced GluN1 expression in hippocampal homogenates (Figure 2, A and B) but not in the P2 (synaptosome) fractions (Figure 2, C and D). GluN2A and GluN2B expression levels in both the total and P2 fractions of the hippocampal homogenates did not differ between WT and PKU mice. Consistent with the normal expression levels of NMDAR subunits in the P2 fraction, there were no changes in the AMPA-NMDA ratios and AMPAR-mediated synaptic transmission at the SC-CA1 synapse in *Pah^Enu2^* mice (Figure 2, E−G, and Supplemental Figure 2). In addition, the magnitude of I_NMDA_ measured in the absence and presence of PEAQX and AV-5 did not differ between WT and *Pah^Enu2^* mice (Figure 2, H and I). Collectively, these results indicate that excitatory synaptic transmission in *Pah^Enu2^* mice is normal in the absence of Phe.

We examined whether Phe-induced hyperactivation of GluN2B affects tonic inhibition in PKU mice, based on the evidence that overexpression and suppression of GluN2B in cultured hippocampal neurons results in decreased and increased tonic inhibition, respectively, through the regulation of trafficking of the GABA_A_R α5 subunit (α5-GABA_A_R) (40). However, we did not detect any differences in the hippocampal expression levels of α5-GABA_A_R (Figure 2J) or tonic GABA currents in CA1 neurons between WT and PKU mice (Figure 2, K and L). In addition, l-Phe (250 μM) perfusion did not affect tonic GABA currents in the CA1 neurons of either genotype. These observations indicated normal tonic inhibition in the brains of *Pah^Enu2^* mice.

### l-Phe impairs synaptic plasticity through the hyperactivation of GluN2B-containing NMDARs.

**Figure 3:**
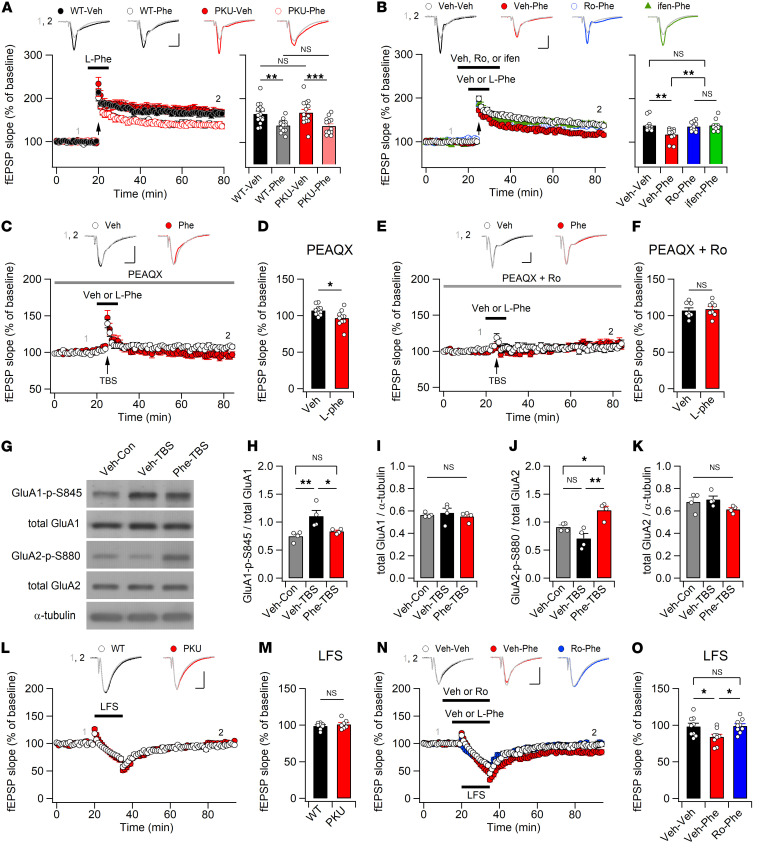
l-Phe perturbs synaptic plasticity through the activity-dependent downregulation of AMPARs. (**A**) l-Phe reduces the magnitude of LTP in both WT and *Pah^Enu2^* mice. Top: representative traces of fEPSP obtained at the indicated time points. Bottom left: fEPSP slopes were normalized to those obtained in the baseline and plotted against time. l-Phe was perfused from 5 minutes before to 1 minutes after TBS (arrow). Bottom right: fEPSP slopes during the last 10 minutes were normalized to baseline. (**B**) GluN2B antagonists block the effect of l-Phe on the TBS (arrow)-induced LTP. Sample traces (top), time course of fEPSP slopes (bottom left), and the magnitude of LTP (bottom right) in each condition. (**C** and **D**) PEAQX blocks LTP induction, and l-Phe perfusion during the peri-TBS period induces an LTD-like decrease in fEPSP slopes. (**E** and **F**) l-Phe and TBS had no effect on the slope of fEPSPs under GluN2A and GluN2B inhibition. (**G**−**K**) Representative Western blots (**G**), and the ratios of phosphorylated GluA1-S845 to total GluA1 (**H**), total GluA1 to -tubulin (**I**), phosphorylated GluA2-S880 to total GluA2 (**J**), and total GluA2 to -tubulin (**K**) in the CA1 region of acute hippocampal sections harvested 30 minutes after TBS. (**L**) WT and *Pah^Enu2^* sections exhibit similar synaptic responses to LFS. (**M**) Normalized fEPSP slopes during the last 10 minutes in WT and *Pah^Enu2^* sections. (**N**) Ro blocks the effect of l-Phe on LTD facilitation. Sample traces of fEPSPs (top). (**O**) Normalized fEPSP slopes during the last 10 minutes in each condition. (**A**−**C**, **E**, **L**, and **N**) Scale bars: 5 ms and 0.5 mV. Statistical analysis was performed using Student’s *t* test (**D**, **F**, and **M**), 1-way (**H**−**K**, and **O**) or 2-way ANOVA (**A**), or Kruskal-Wallis test (**B**) with post hoc Tukey’s test. *P < 0.05, **P < 0.01, ***P < 0.001, and NS, *P* ≥ 0.05. Con, control; Veh, vehicle.

To examine synaptic plasticity in the hippocampus of *Pah^Enu2^* mice, we measured the field excitatory postsynaptic potentials (fEPSPs) at the SC-CA1 synapses. Interestingly, 4 episodes of theta-burst stimulation (TBS) of SC axons induced similar magnitudes of long-term potentiation (LTP) in both genotypes (Figure 3A), indicating normal LTP in *Pah^Enu2^* mice in the absence of Phe. Next, we investigated the effects of Phe concentration in the PKU CSF on hippocampal synaptic plasticity. Surprisingly, however, perfusion of l-Phe (250 μM) during the TBS significantly attenuated LTP induction (Figure 3A), even though l-Phe had no effect on the fiber volley amplitudes, fEPSP slopes, or population spikes in the basal condition (Supplemental Figure 3). Moreover, the extent of LTP attenuation by l-Phe perfusion did not differ between the WT and *Pah^Enu2^* sections (Figure 3A). Consistent with I_NMDA_ facilitation at a concentration of 10 μM glycine (Figure 1I), l-Phe-induced attenuation of LTP was observed under treatment with 10 μM glycine in the ACSF (Supplemental Figure 4, A and B).

We hypothesized that CSF Phe hyperactivates extrasynaptic GluN2B-containing NMDARs during TBS, thereby inducing the homeostatic downregulation of AMPARs (41). Indeed, treatment of sections with Ro (2 μM) or ifen (6 μM) completely blocked the effect of l-Phe on LTP attenuation in the WT sections (Figure 3B). In the absence of Phe, Ro and ifen had no effect on TBS-induced LTP (Supplemental Figure 4, C and D). Meanwhile, PEAQX blocked LTP induction, such that the increased fEPSPs caused by TBS rapidly returned to baseline levels (Figure 3C). Under these conditions, perfusion of l-Phe during TBS induced a long-term depression–like (LTD-like) decrease in synaptic strength (Figure 3, C and D), indicating the downregulation of AMPARs. Consistent with this, neither LTP nor l-Phe–induced synaptic depression was observed after TBS when the sections were cotreated with PEAQX and Ro (Figure 3, E and F).

We subsequently investigated the molecular changes in AMPARs associated with l-Phe–induced perturbations during LTP (Figure 3, G−K). TBS of the SC axons induced phosphorylation of serine 845 on the GluA1 subunit (GluA1-S845) without significantly affecting the phosphorylation status of serine 880 on the GluA2 subunit (GluA2-S880). However, l-Phe perfusion during TBS suppressed GluA1-S845 phosphorylation but significantly enhanced GluA2-S880 phosphorylation in the CA1 area of hippocampal sections (Figure 3, G−K). Considering that LTP and LTD stimuli bidirectionally modulate GluA1-S845 phosphorylation and that GluA2-S880 phosphorylation promotes AMPAR endocytosis (42, 43), the attenuated GluA1-S845 phosphorylation and enhanced GluA2-S880 phosphorylation suggest that enhanced AMPAR endocytosis may underlie the attenuated LTP caused by l-Phe perfusion.

We further determined the synaptic response of *Pah^Enu2^* hippocampus to low-frequency stimulation (LFS), which is widely used to induce LTD in juvenile mice. Although LFS (1 Hz; *n* = 900 stimulations) did not induce LTD in either genotype, the fEPSP slopes for WT and *Pah^Enu2^* mice decreased to the same extent during LFS and gradually returned to baseline levels (Figure 3, L and M). Notably, l-Phe perfusion promoted synaptic depression during LFS and induced stable LTD, which was blocked by Ro perfusion during LFS (Figure 3, N and O). Collectively, these results suggest the concentration of Phe observed in PKU CSF hyperactivates GluN2B-containing receptors and induces the activity-dependent downregulation of AMPARs.

### l-Phe challenge decreases neural activity.

**Figure 4:**
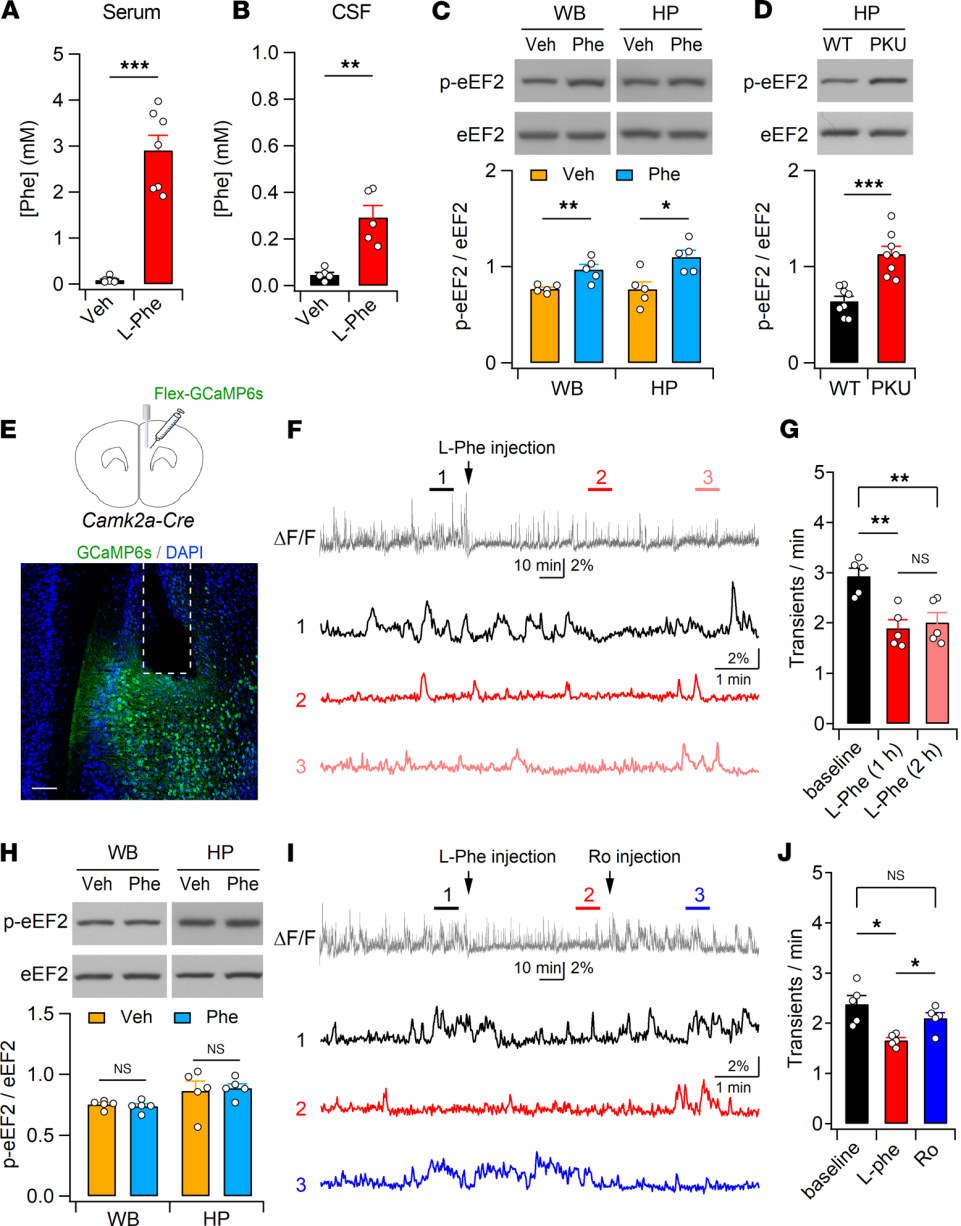
l-Phe loading decreases neural activity in adult mice. (**A** and **B**) Phe concentrations in the serum (**A**) and CSF (**B**) were measured 30 minutes after vehicle (Veh) or l-Phe (1 mg/g, i.p.) administration. (**C**) Western blot analyses for the protein levels of p-eEF2 and eEF2 in Veh- and l-Phe–treated mice. Whole brains (WB) and hippocampi (HP) were collected 30 minutes after Veh or l-Phe (1 mg/g, i.p.) administration. Bottom: Quantification of the p-eEF2 to eEF2 ratio. (**D**) Enhanced eEF2 phosphorylation (top) and increased p-eEF2 to eEF2 ratio (bottom) in the *Pah^Enu2^* hippocampus. (**E**) Experimental design for fiber photometry recording in the mPFC of *Camk2a-Cre* mice. Bottom: immunohistochemical staining of an mPFC section showing the GCaMP6s-expressing cells (green) and canula placement. DAPI (blue) was used to identify the brain structures. Calibration, 200 μm. (**F**) Neuronal activity of CaMKII-expressing cells in the mPFC was decreased by l-Phe administration. The bottom panel shows the ΔF/F signals obtained during the indicated periods (1, 2, and 3) on an expanded time scale. (**G**) Quantification of the frequency of Ca^2+^ transients obtained from 5 mice. (**H**) Ro blocks l-Phe–induced eEF2 phosphorylation in the hippocampus. Ro (3 mg/kg, i.p.) was administered 30 minutes before l-Phe or vehicle injection. (**I**) l-Phe and Ro were sequentially administered to *Camk2a-Cre* mice during the recording. (**J**) Summary of the frequency of F transients during the baseline and perfusion of l-Phe and Ro. Statistical analysis was performed using the Mann-Whitney *U* test (**A**), Student’s *t* test (**B**−**D** and **H**), or 1-way ANOVA with post hoc Tukey’s test (**G** and **J**). *P < 0.05, **P < 0.01, ***P < 0.001, and NS, *P* ≥ 0.05.

Oral Phe loading shifted the dominant peak of the EEG background activity to a lower frequency spectrum in _ETP_PKU (26). However, the effect of Phe elevation on neural activity remains unclear. Similar to previous reports that observed elevation of Phe_Serum_ and Phe_Brain_ in rodents after i.p. administration of l-Phe (44, 45), i.p. l-Phe administration to WT mice at a dose of 1 mg/g body weight elevated Phe_Serum_ and Phe_CSF_ levels comparable to those of PKU mice (Figure 4, A and B). As the phosphorylation status of eukaryotic elongation factor 2 (eEF2) changes rapidly in response to neuronal activity (46), we examined the effect of l-Phe challenge on the proportion of phosphorylated eEF2 (p-eEF2) in total eEF2. Mice treated with l-Phe (1 mg/g) had an increased p-eEF2 to eEF2 ratio in both the whole brain and hippocampus compared with vehicle-treated mice (Figure 4C). Enhanced eEF2 phosphorylation was further detected in the *Pah^Enu2^* hippocampus (Figure 4D), indicating that both *Pah^Enu2^* and l-Phe–treated mice had reduced neuronal activity in the brain.

To directly monitor changes in neuronal activity induced by l-Phe challenge, we performed fiber photometry recordings in freely behaving mice and observed a reduction in neuronal activity in the medial prefrontal cortex (mPFC) of *Camk2a-Cre* mice after l-Phe administration (Figure 4, E–G). The results indicate that l-Phe challenge modifies neuronal activity in WT mice similar to that of the *Pah^Enu2^* mice. Intriguingly, pretreatment (30 minutes before l-Phe injection) with Ro abolished the effect of l-Phe on eEF2 phosphorylation in both the whole brain and hippocampus (Figure 4H), indicating an association between GluN2B receptor activation and the l-Phe–induced reduction in neuronal activity. In support of this idea, fiber photometry detected a reduction in and recovery of neuronal activity in the mPFC of *Camk2a-Cre* mice after l-Phe and subsequent Ro injections (Figure 4, I and J). These results suggest elevated Phe levels decrease neural activity via GluN2B-dependent mechanisms.

### l-Phe challenge recapitulates cognitive symptoms of PKU in adult mice.

*Pah^Enu2^* mice exhibit different behavioral phenotypes depending on their genetic background, despite similar biochemical phenotypes (47–50). Consistent with previous studies (47, 51), we observed impaired learning and memory in *Pah^Enu2^* mice with a C57BL/6N background. Although *Pah^Enu2^* mice behaved normally in the open-field test (OFT) and the visible platform version of the Morris water maze (MWM) test, they displayed impaired performance in the novel object recognition (NOR), object location memory (OLM), and hidden platform version of the MWM tests (Supplemental Figure 5).

**Figure 5:**
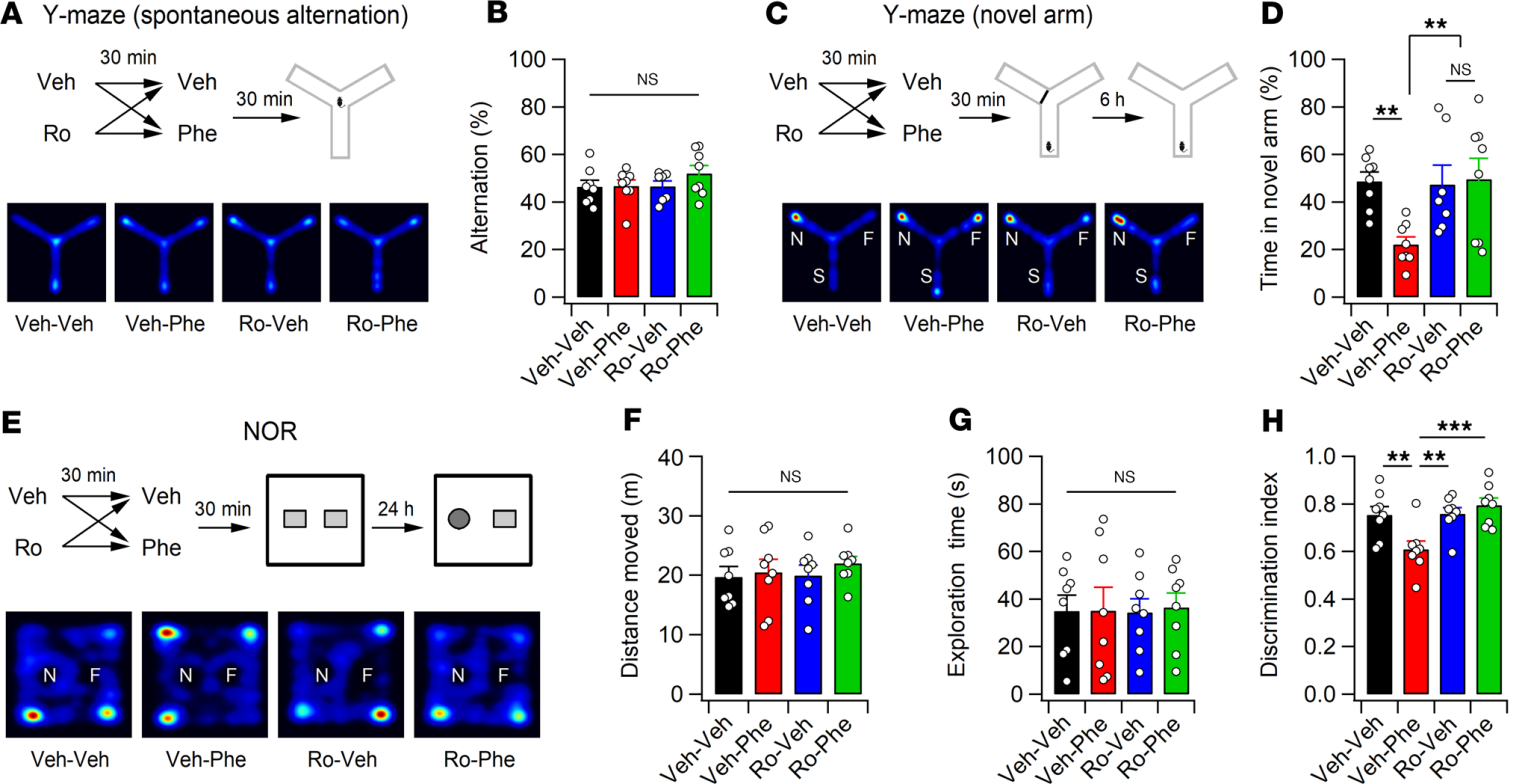
l-Phe loading impairs learning in mice. (**A**) Experimental design (top) and activity paths (bottom) of mice in the Y maze. (**B**) The percentage of spontaneous alternation in the Y maze measured 30 minutes after l-Phe or vehicle injections. (**C**) Mice received l-Phe or vehicle 30 minutes before training (top). Bottom: activity paths of mice during the test session. The test session was conducted 6 hours after the training session. N, novel arm; F, familiar arm; S, start arm. (**D**) Mice treated with l-Phe spent significantly less time in the novel arm. (**E**) Ro and l-Phe were administered 1 hour and 30 minutes before the training session of NOR, respectively. Bottom: activity paths of mice during the test session of NOR. F, familiar object; N, novel object. (**F** and **G**) Quantification of the distance moved (**F**) and time spent exploring the 2 objects (**G**) during the test session of NOR. (**H**) Relative preference for the novel object was calculated using a discrimination index. Statistical analysis was performed using 2-way ANOVA with post hoc Tukey’s test (**B**, **D,** and **F**−**H**). **P < 0.01, ***P < 0.001, and NS, *P* ≥ 0.05.

We hypothesized that if Phe_CSF_ exerts a profound effect on cognitive dysfunction in *Pah^Enu2^* mice, l-Phe challenge would induce similar phenotypes in adult WT mice. l-Phe challenge did not affect spontaneous alternation of WT mice during the Y-maze test, indicating normal working memory (Figure 5, A and B). However, l-Phe treatment 30 minutes before the training phase of novel arm exploration significantly impaired novel arm discrimination in mice during the test phase, which was performed 6 hours after the training phase (Figure 5, C and D). Similarly, mice that received l-Phe 30 minutes before the sample phase of the NOR test spent less time exploring the novel object than did vehicle-treated mice in the test session, which was performed 24 hours after the sample phase (Figure 5, E−H). Importantly, pretreatment (30 minutes before l-Phe administration) of mice with Ro (3 mg/kg) abolished the l-Phe–induced impairment in novel arm discrimination and NOR (Figure 5, C−H). We confirmed that the doses of l-Phe and Ro used in this study did not affect general activity or exploratory behaviors of mice in the OFT (Supplemental Figure 6). Collectively, these results suggest elevated Phe is sufficient to impair learning and memory, and that GluN2B receptors play a key role in l-Phe-induced cognitive dysfunction.

### Suppression of GluN2B rescues impaired learning in Pah^Enu2^ mice.

**Figure 6:**
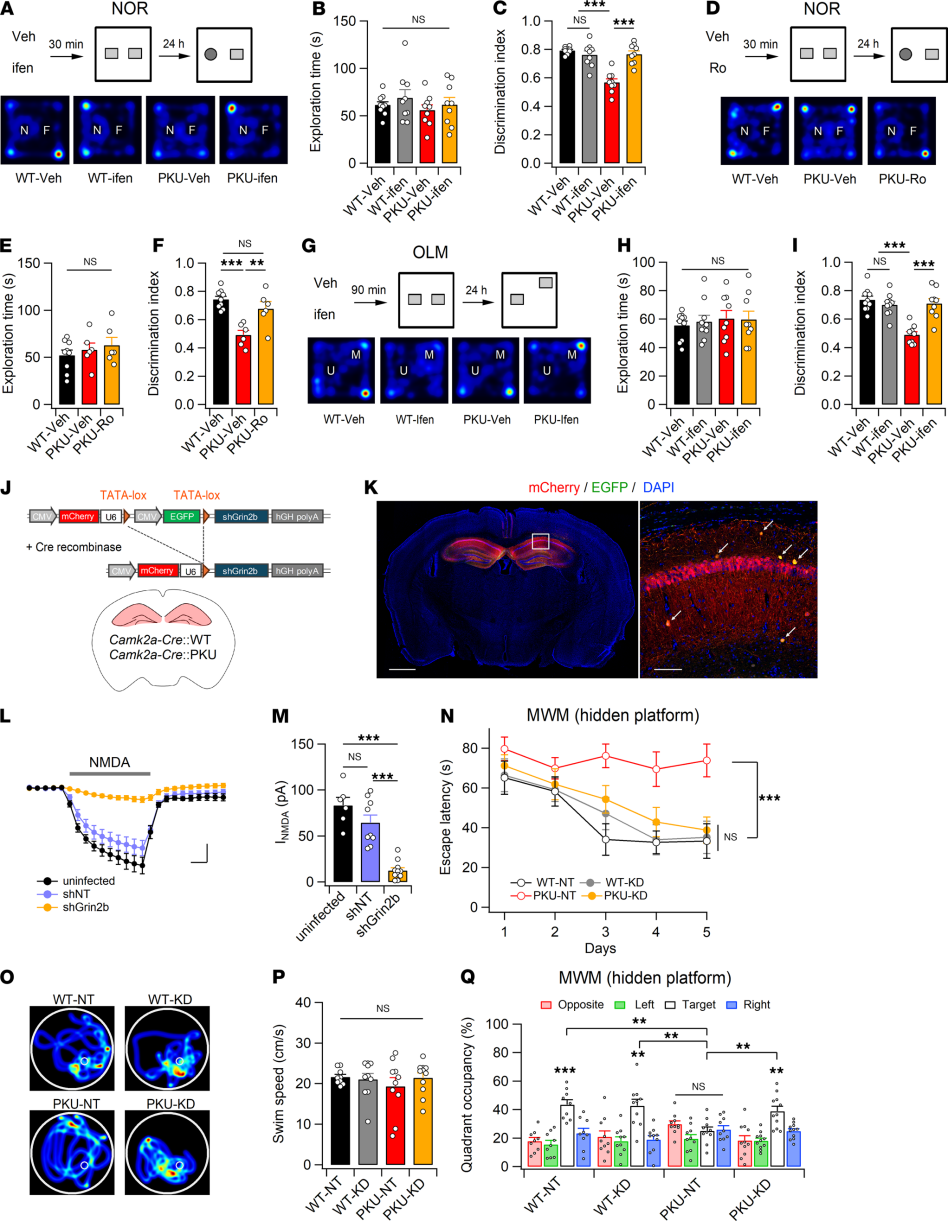
GluN2B suppression improves learning and memory impairment in *Pah^Enu2^* mice. (**A**) Mice were treated with vehicle (Veh) or ifen 30 minutes before the NOR training session. Bottom: example path recordings of mice during the NOR test session. F, familiar object; N, novel object. (**B** and **C**) Time spent exploring the 2 objects (**B**) and preference for novel object (**C**). (**D**−**F**) Same as **A**−**C** but for Ro. (**G**) Experimental design (top) of the OLM test and sample path recordings (bottom) of mice during the OLM test session. M, moved object; U, unmoved object. (**H** and **I**) Time spent exploring the 2 objects (**H**) and preference for the moved object (**I**) during the OLM test session. (**J**) Design of the Cre-dependent expression of shGrin2b in CaMKIIα-expressing cells in the dorsal hippocampus. (**K**) Immunohistochemical staining of a hippocampal section showing the expression of mCherry in the hippocampus. Calibration, 1 mm. Right, magnified image of the region corresponding to the white box in the left panel showing the absence of an EGFP signal in CA1 principal cells. Yellow cells (arrows) indicate putative interneurons expressing both EGFP and mCherry. Calibration, 100 μm. (**L**) I_NMDA_ in the CA1 neurons was measured in the presence of blockers for GluN2A, Na^+^-channels, AMPARs, and GABA_A_Rs. Scale bars: 2 minutes and 20 pA. (**M**) Reduced I_NMDA_ in CA1 pyramidal neurons expressing shGrin2b. (**N**) Escape latency of mice during the 5-day training session of the MWM test. (**O**−**Q**) Representative swim path (**O**), swim speed (**P**), and quadrant occupancy (**Q**) of mice during the MWM probe trials. Statistical analysis was performed using 1-way (**E**, **F**, **M**, and **Q**) or 2-way (**B**, **C**, **H**, **I**, **N**, and **P**) ANOVA with post hoc Tukey’s test. **P < 0.01, ***P < 0.001, and NS, *P* ≥ 0.05.

Because excitatory synaptic transmission and plasticity in *Pah^Enu2^* hippocampal sections did not differ from those in WT sections in normal ACSF, we wondered whether suppressing GluN2B would restore impaired learning and memory in adult *Pah^Enu2^* mice. We examined the effects of GluN2B antagonists on the NOR performance of *Pah^Enu2^* mice and found that administration of ifen (5 mg/kg, i.p.) or Ro (3 mg/kg, i.p.) 30 minutes before the sample session significantly improved the NOR performance in *Pah^Enu2^* mice during the test session (Figure 6, A−F). Consistent with this observation, Ro administration increased the activity of CaMKII-expressing neurons in the *Camk2a-Cre*;*Pah^Enu2^* mPFC, as detected by fiber photometry (Supplemental Figure 7). The administration of ifen also improved OLM in PKU mice (Figure 6, G−I), further confirming the effect of GluN2B suppression on learning performance in PKU mice. Additionally, ifen did not induce abnormal behavior in WT or *Pah^Enu2^* mice during the OFT (Supplemental Figure 8).

We wondered whether GluN2B suppression could rescue impaired MWM performance in *Pah^Enu2^* mice. To stably suppress the function of GluN2B receptors, we generated an adeno-associated virus (AAV) carrying a Cre-dependent shRNA expression vector that targets GluN2B (Figure 6J). Infection of the shGrin2b virus into the dorsal hippocampus of *Camk2a-Cre* mice resulted in a significant reduction in GluN2B expression, without affecting GluN1, GluN2A, or PSD-95 levels in the synaptosomal fraction (Figure 6K and Supplemental Figure 9). We further confirmed a significant reduction in the GluN2B-mediated current in CA1 pyramidal neurons infected with the shGrin2b virus compared with those infected with the nontargeting scrambled sequence or uninfected (Figure 6, L and M).

Infection of the dorsal hippocampus with shGrin2b did not significantly affect MWM performance in adult *Camk2a-Cre* mice. *Camk2a-Cre*;*Pah^Enu2^* mice infected with shGrin2b exhibited significantly enhanced learning during the 5-day training period, whereas *Camk2a-Cre*;*Pah^Enu2^* mice expressing scrambled sequences did not show any signs of learning during the same period (Figure 6N). Consistent with this, *Camk2a-Cre*;*Pah^Enu2^* mice expressing shGrin2b exhibited quadrant occupancy levels similar to those of *Camk2a-Cre* mice during the probe trial (Figure 6, O−Q). Collectively, these results suggest that GluN2B suppression improves cognitive function in adult PKU mice.

## Discussion

In this study, we investigated the synaptic mechanisms underlying cognitive impairment associated with PKU. Our results provide evidence that Phe predominantly affects GluN2B-containing, rather than GluN2A-containing, NMDARs. Importantly, we found that the concentration of Phe in the CSF of patients with PKU, in contrast to that observed in the serum, upregulated GluN2B receptor activity. GluN2B receptors are located predominantly, but not exclusively, in the extrasynaptic area of the mature synapses (32, 52), and the activity of glutamate transporters limits the activation of extrasynaptic NMDARs during low-frequency activity (53). However, bursting or sustained synaptic activity results in glutamate spillover, which activates extrasynaptic NMDARs (54). Phe present in PKU CSF may induce hyperactivation of GluN2B-containing NMDARs and consequently lead to abnormal temporal integration of synaptic inputs and activity-dependent downregulation of AMPARs (41, 52).

Based on the finding that l-Phe, at concentrations observed in or higher than that in PKU serum, reduces I_NMDA_ in cultured hippocampal neurons (21, 22), it is widely believed that elevated Phe in PKU inhibits NMDAR function. Although the Phe concentration is higher than 1 mM in the serum, clinical studies have shown that the Phe concentration in PKU CSF is much lower than that in the serum, because Phe crosses the BBB through l-type amino acid transporter 1–mediated transport (3). Phe_Blood_ of 1.0 mmol/L were consistently associated with Phe_brain_ of 0.2–0.3 mmol/kg in humans (25), and the linear regression model predicts the relationship between Phe_brain_ and Phe_Blood_ as follows (in μM): Phe_Brain_ = 22.02 + 0.22 × Phe_Blood_ (28). A recent preclinical study further reported a strong correlation between Phe_brain_ and Phe_Plasma_ in PKU (*Pah^enu2^*) mice and that Phe_brain_ in the C56BL/6 and BTBR strains of PKU mice were 0.298 and 0.392 mmol/kg, respectively (29). Consistent with this, we observed that Phe_CSF_ and Phe_Serum_ in the C56BL/6N strain of *Pah^enu2^* mice were 0.33 ± 0.03 and 1.91 ± 0.21 mM, respectively, mean ± SEM (Figure 1).

Synaptic NMDARs in the CNS undergo subunit replacement, predominantly from GluN2B to GluN2A, during early postnatal development (52, 55). At immature glutamatergic synapses, GluN2B contributes to synaptic NMDAR signaling, whereas the activity of GluN2B negatively regulates AMPAR incorporation (56, 57). Hence, in contrast to the adult brain, elevated Phe levels may enhance the electrical and biochemical signaling of synaptic NMDARs in the neonatal brain. Hyperactivation of synaptic GluN2B may delay the development of glutamatergic synapses and brain maturation by hampering the incorporation of AMPARs into immature synapses. Untreated PKU results in severe intellectual impairment, and late treatment with a low-Phe diet partially reverses this cognitive impairment (2, 58), indicating that the neonatal brain, in which GluN2B is predominant (55), is more sensitive to elevated Phe levels than is the mature brain. A recent study reported that the overexpression of GluN2B reduces both tonic GABA currents and the surface expression of α5-GABA_A_R in hippocampal neurons (40). Although the expression levels of α5-GABA_A_R and epileptic mechanisms are unknown in patients with PKU, epilepsy is frequently accompanied by PKU. Adult (aged 18−20 weeks), but not younger (aged 5−7 weeks), *Pah^Enu2^* mice of the BTBR strain also have enhanced susceptibility to audiogenic seizures compared with WT mice (59). However, in the present study, the hippocampal expression levels of α5-GABA_A_R and tonic GABA currents in CA1 pyramidal neurons in *Pah^Enu2^* mice did not differ from those in WT mice. In addition, spontaneous seizure behavior was not observed in the *Pah^Enu2^* mice.

Similar to our results, amyloid-β protein (Aβ) has been suggested to hyperactivate extrasynaptic GluN2B receptors and consequently facilitate LTD, but impair LTP, in the hippocampus (60, 61). Moreover, treatment of cultured neurons with Aβ enhances GluA2-S880 phosphorylation (62) but suppresses GluA1-S845 phosphorylation induced by chemical LTP stimulation (63). These findings indicate that shared mechanisms, comprising hyperactivation of extrasynaptic NMDARs and perturbed synaptic plasticity, underlie the cognitive impairment in PKU and Alzheimer’s diseases. l-Phe is unlikely to inhibit glutamate reuptake or promote glutamate spillover but acts directly on GluN2B receptors, because l-Phe did not affect NMDAR-EPSCs in the absence of TBOA, and l-Phe increased I_NMDA_ in HEK293 cells expressing GluN1 and GluN2B. In addition to possible structural changes in the brain (19), perturbed synaptic plasticity induced by hyperactivation of GluN2B may contribute to cognitive deficits in adults with PKU. A recent clinical study reported that early treated adults with PKU underperformed on cognitive tests, including processing speed, executive function, and learning; processing speed was significantly related to Phe concentration at the time of testing (5). In addition, early treated adults with lower Phe levels performed better than those with higher Phe levels in most cognitive domains, including IQ (6). These observations indicate that elevated Phe levels still influence cognitive function in adults (19) and that dietary control alone may not be sufficient to prevent suboptimal cognitive outcomes (5). Tetrahydrobiopterin and large neutral amino acid (LNAA) treatments are known to have beneficial effects on cognitive functions (2). LNAA supplementation reduces brain Phe through competition for transport across the BBB, improves brain serotonin and norepinephrine concentrations, and increases tyrosine hydroxylase and tryptophan hydroxylase activities, which are inhibited by excessive brain Phe concentrations (64). However, a substantial unmet need exists for patients with PKU. Notably, CSF analyses revealed a significant enhancement in the levels of Aβ_1-42_, total tau, and phosphorylated tau in early-treated patients with PKU compared with healthy control study participants (19). These observations further support the therapeutic potential of GluN2B inhibitors for the treatment of cognitive complications associated with PKU.

A previous study reported that Phe-reducing treatments, dietary Phe restriction, or liver-directed gene therapy with a Pah-expressing recombinant AAV vector, for 8−10 weeks starting at 8 weeks old, did not improve the performance of C57BL/6-*Pah^Enu2^* mice on the visible platform version of the MWM test but did correct CNS dopamine and serotonin deficiencies (51). Because *Pah* KO mice exhibit progressive ophthalmic pathology characterized by hypermature cataract at 2.5−6 months of age (65), it is possible that visual impairment might have affected the MWM performance of *Pah^Enu2^* mice at 16–18 weeks of age (51). Meanwhile, liver-directed gene therapy at 3 weeks of age reversed spatial learning deficits and biochemical abnormalities in BTBR-*Pah^Enu2^* mice at 2 and 6 months after Pah-containing adenoviral vector administration (50). Intriguingly, both untreated and treated BTBR-*Pah^Enu2^* mice, 2 and 6 months old, performed normally on the visible platform training (50). We further observed that C57BL/6N-*Pah^Enu2^* mice at 8−12 weeks of age were not impaired in finding the visible platform despite elevated Phe levels in both the CSF and blood, but they exhibited profound impairments in finding the hidden platform.

One important finding of this study is that *Pah^Enu2^* mice exhibit normal synaptic transmission and plasticity in the absence of Phe. The reversal of impaired learning and memory in adult *Pah^Enu2^* mice by both pharmacological treatment and shRNA-mediated gene silencing further indicated that elevated Phe levels in the CSF, rather than irreversible brain damage, profoundly affected learning and memory in *Pah^Enu2^* mice. In support of this idea, the acute administration of l-Phe to adult WT mice was sufficient to induce impairments in NOR and OLM performance, which were abrogated by pretreatment with GluN2B inhibitors (Figure 5). A single administration of l-Phe or GluN2B inhibitors during behavioral testing is unlikely to induce or restore the structural and neurochemical abnormalities observed in the PKU brain. However, our results did not suggest that brain dysfunction in PKU, including cognitive impairment and increased rates of depression, anxiety, psychosis, and epilepsy, is solely attributable to elevated Phe levels and the resultant synaptic disturbances. In addition to elevated Phe levels, reduced protein synthesis and WMAs, as well as decreased concentrations of dopamine, serotonin, and LNAAs, have been observed in the brains of PKU mice and patients with PKU (16–18, 66). However, the cognitive consequences of these changes are not fully understood. Further investigations are required to elucidate the effects of diverse structural and neurochemical changes in the brain on the cognitive function of patients with PKU.

## Methods

### Sex as a biological variable.

Our study was performed using C57BL/6N mice of both sexes, except for the l-Phe challenge, which was performed exclusively using male mice. No sex-dependent differences in biological outcomes were observed in either WT or *Pah^Enu2^* mice; therefore, the results of the l-Phe challenge experiments were also expected to be relevant to female mice.

### Animals.

C57BL/6N mice (Orient Bio) were group-housed (*n* = 3−5/cage) in a specific pathogen-free facility and maintained in a climate-controlled room under a 12 h light/dark cycle. All mice had free access to water and standard chow (20% protein containing 0.98% l-Phe). *Pah^Enu2^* mice (Jackson Laboratory; catalog 002232) were provided by Sung-Chul Jung (Ewha Womans University) and backcrossed with C57BL/6N mice for at least 5 generations before use. Genotypes of the *Pah^Enu2^* mice were determined by restriction endonuclease digestion of the PCR product. Genomic DNA was extracted from mouse tail, and exon 7 of the *Pah* gene was amplified using oligonucleotide primers 5′-CCTTGGGGAGTCATACCTCA-3′ and 5′- ATAAAGCAGGCAGTGGATCA-3′. The 317-bp PCR product was digested with the restriction endonuclease MboII overnight at 37ºC, and the restriction fragments were separated by electrophoresis on a 2% acrylamide gel. *Camk2a-Cre* transgenic mice were provided by Yong-Seok Lee (Seoul National University) and were backcrossed with C57BL/6N mice for at least 10 generations prior to use.

### Measurement of Phe concentration.

To obtain mouse serum and CSF, mice (6−9 weeks old) were deeply anesthetized with a mixture of zoletil (50 mg/kg, i.p.) and xylazine (1 mg/kg, i.p.). Blood samples (100−200 μL) were collected into capillary tubes by retro-orbital bleeding, transferred into a microcentrifuge tube, and kept for 1 hour at room temperature to allow the blood to clot. The blood was centrifuged at 1,500*g* for 15 minutes at 4°C, and serum was carefully collected using a micropipette and stored at –80ºC.

To collect CSF from the cisterna magna, a sharpened glass capillary was pulled using a micropipette puller (catalog PC-100; Narishige), and attached to a 3-way valve with a syringe via an aspirator tube. Anesthetized mice were placed in a stereotaxic device with the nose pointed down at approximately 45°. The skin was then cut over the midline, and muscles were removed to expose the cisterna magna. The atlanto-occipital membrane above the cisterna magna was carefully removed with a cotton swab. A sharpened glass capillary was gently inserted into the cisterna magna between the blood vessels. The clear liquid (10−15 μL) collected in the glass capillary flowed into a collection tube and then was centrifuged and stored at –80ºC.

Serum and CSF Phe concentrations were measured using a Phe assay kit (catalog KA3781; Abnova) according to the manufacturer’s instructions. Briefly, the serum and CSF were diluted 1:20 and 1:5, respectively, to ensure the Phe concentrations in the samples were within the detection range (2−300 μM) of the kit. The diluted samples and standard Phe solutions were mixed with the working reagents, and the mixture was incubated for 20 minutes at room temperature in the dark. The fluorescence intensity (F) at λ_Ex_/_Em_ = 530/580 nm was measured using a Spark multimode microplate reader (TECAN). Phe concentrations in the samples were calculated using the following formula: [l-Phe] = (F_Sample_ − F_Blank_) / slope (μM), where F_Sample_ and F_Blank_ represent the F values of the sample and sample blank, respectively. The slope was derived from the l-Phe standard curve.

### Electrophysiology.

Parasagittal hippocampal sections (400 μm thick) were prepared from 6- to 9-week-old mice using a vibratome (Leica) in ice-cold dissection buffer (sucrose 230 mM; NaHCO_3_ 25 mM; KCl 2.5 mM; NaH_2_PO_4_ 1.25 mM; d-glucose 10 mM; Na-ascorbate 1.3 mM; MgCl_2_ 3 mM; CaCl_2_ 0.5 mM, pH 7.4 with 95% O_2_/5% CO_2_). Immediately after sectioning, the CA3 region was surgically removed. The sections were allowed to recover at 36°C for 1 hour in normal ACSF (NaCl 125 mM; NaHCO_3_ 25 mM; KCl 2.5 mM; NaH_2_PO_4_ 1.25 mM; d-glucose 10 mM; MgCl_2_ 1.3 mM; CaCl_2_ 2.5 mM, pH 7.4 with 95% O_2_/5% CO_2_), and then maintained at room temperature.

Sections were placed in a submerged recording chamber, which was perfused continuously with heated (29°C–30°C) ACSF. All electrophysiological recordings were performed using a MultiClamp 700B amplifier and Digidata 1440A interface (Molecular Devices). The signals were filtered at 2.8 kHz and digitized at 10 kHz. Data were analyzed using custom macros written in Igor Pro (WaveMetrics).

To measure I_NMDA_ in CA1 neurons or HEK293 cells at a holding potential of –40 mV, whole-cell voltage clamp recordings were made using patch pipettes (3–4 MΩ) filled with solution containing (in mM) 100 Cs-gluconate, 10 TEA-Cl, 10 CsCl, 8 NaCl, 10 HEPES, 4 Mg-ATP, 0.3 Na-GTP, 0.5 QX-314-Cl, and 10 EGTA, adjusted to pH 7.25 and 290 mOsm/kg. During I_NMDA_ recordings in CA1 neurons, picrotoxin (50 μM), NBQX, and TTX (1 μM) were added to the ACSF. NMDAR-EPSCs were measured using the same pipette solution used for measurement of I_NMDA_. Picrotoxin and NBQX were added to ACSF. Synaptic responses were evoked at 0.05 Hz with an ACSF-filled broken glass pipette (0.3–0.5 MΩ) placed in the proximal region of the stratum radiatum. The series and seal resistances were continuously monitored using short (50 ms) test (2 mV) pulses, and the data were discarded if they changed by more than 20% during the recordings.

To measure the synaptic AMPA to NMDA ratio, AMPAR-mediated EPSCs were obtained by averaging 30–40 traces recorded at –70 mV. The stimulation intensity was adjusted to yield a 100–300 pA EPSC peak amplitude. After recording the AMPAR-mediated EPSCs, NBQX was added to the ACSF and 30–40 traces of NMDAR-mediated EPSCs were recorded at +40 mV.

Tonic GABA currents were measured at –70 mV using a pipette solution containing (in mM) 140 CsCl, 8 TEA-Cl, 10 HEPES, 4 Mg-ATP, 0.3 Na-GTP, 0.5 QX-314-Cl, and 10 EGTA, adjusted to pH 7.25 and 290 mOsm/kg. NBQX and AP-5 were present in ACSF throughout the recordings. Tonic GABA currents were calculated as the difference in the mean baseline current during and before bicuculline application.

To record fEPSPs at SC-CA1 synapse, a recording (3−4 MΩ) pipette filled with ACSF was placed in the stratum radiatum. Synaptic responses were evoked by stimulating SCs with an ACSF-filled broken glass pipette (0.05 Hz), and the stimulation intensity was adjusted to yield approximately 30% of the maximal responses. LTP was elicited by 4 trains of TBS, with a 10 second intertrain interval. The TBS consisted of 10 bursts, each comprising 4 pulses at 100 Hz, with an interburst interval of 200 ms. Sections displaying unstable (10%) baseline recordings were excluded from the analysis. For Western blot analysis of AMPARs in the CA1 region (Figure 3G), the CA1 regions were surgically dissected from hippocampal sections 30 minutes after TBS, and the CA1 sections were rapidly frozen using liquid nitrogen before being stored at −80ºC until subsequent use. LFS consisted of 900 pulses at 1 Hz. Extracellular population spike recordings were performed by placing a recording electrode in the CA1 stratum pyramidale. Synaptic responses were evoked by a stimulating electrode placed in the stratum radiatum.

### Cell culture and transfection.

Cells of the human embryonic kidney cell line (HEK 293T/17; American Tissue Culture Collection; catalog CRL-11268) were cultured on poly-d-lysine–coated glass coverslips in high-glucose (4.5 g/L) Dulbecco’s modified Eagle’s medium supplemented with 4 mM l-glutamine, 3.7 g/L sodium bicarbonate, 10% (v/v) fetal bovine serum, 1 mM sodium pyruvate, and 1% (v/v) penicillin/streptomycin. After transfection, the cell culture medium was replaced with fresh medium containing D-AP5 (50 μM) and 1 mM MgCl_2_. cDNA vectors expressing untagged human GluN1-1a, GluN2A, and GluN2B were provided by Young Ho Suh (Seoul National University) (67). Plasmids were transfected at the DNA ratio of GRIN1, GRIN2A or GRIN2B, and EGFP at 2:2:1 (total = 0.5 μg/coverslip) using the Lipofectamine 3000 transfection reagent kit (Thermo Fisher Scientific). Transfected cells were identified by EGFP signals, and electrophysiological recordings were performed 24−72 hours after transfection.

### Drugs.

l-Phe was purchased from Sigma-Aldrich. To prepare the stock solution, l-Phe was dissolved to 1 M in 100 mL of distilled water using NaOH at 37°C, and the stock solution (pH 7.4) was aliquoted and cryopreserved until use. All reagents were purchased from Sigma-Aldrich, except for PEAQX (0.5 μM), Ro25-6981 (2 μM), ifen (6 μM), QX-314-Cl (0.5 mM), NBQX (10 μM), TBOA (10 μM), bicuculline (40 μM), and D-AP-5 (50 μM), which were purchased from Hello Bio.

### Behavioral analyses.

Behavioral tests were performed between 10 am and 6 pm in 8- to 12-week-old mice. All experimental mice were acclimated to the behavior testing room for at least 1 hour prior to testing. The testing apparatus was cleaned with 70% ethanol between trials.

The OFT was conducted in open field boxes (40 × 40 × 40 cm) with opaque walls in a dimly lit room. The mice were placed in the center of an open field box, and their behavior was monitored using video recordings. The total distance traveled in the entire box and the time spent in the center zone (20 × 20 cm) were calculated using video tracking software (Ethovision XT; Noldus).

The NOR and OLM tests were conducted in the same box used for the OFT but with 2 identical objects in the middle of the box. During the acquisition (sample) session of the NOR and OLM tests, each mouse was placed in the center of the box and allowed to explore the 2 objects for 10 minutes. The NOR and OLM test sessions were conducted 24 hours after the acquisition session. During the NOR test session, mice were returned to a box in which 1 of the familiar objects was replaced with a new object. To minimize any bias in the location of the objects, the relative locations of familiar and novel objects were counterbalanced between trials. The behavior of each mouse was monitored for 10 minutes using video recordings, and object interaction was defined as sniffing, brief contact, and/or approaching an object. The discrimination index (%) of the NOR test was calculated as follows: [(novel object interaction) / (familiar object interaction + novel object interaction)] × 100. During the OLM test session (10 minutes), the mice were returned to the arena where 1 of the 2 familiar objects was moved to the corner of the box. The position of the moved object was counterbalanced between mice. The discrimination index (%) of the OLM test was calculated as follows: [(moved object interaction) / (unmoved object interaction + moved object interaction)] × 100.

Y-maze tests were conducted in a symmetrical, top-open, Y-shaped maze with acrylic walls. Each arm of the Y maze was 35 cm long × 5 cm wide × 13 cm tall. The mice were allowed to explore all 3 arms for 30 minutes, and spontaneous alternations by each mouse were analyzed from the initial 10 minutes of the habituation period. Spontaneous alternations (%) were defined as consecutive entries into 3 different arms (e.g., ABC or BAC, but not ACA) of the Y maze divided by the number of possible alternations: [number of alternations / (number of total arm entries – 2) × 100]. During the training session of the spatial reference memory test, 1 of the 3 arms was blocked by an acrylic baffle. Each mouse was placed in the starting arm and allowed to freely explore both the starting and other arms for 15 minutes. The test session of the spatial reference memory test was conducted 6 hours after the training session. The acrylic baffle was removed and the mice were allowed to explore all 3 arms of the Y maze for 5 minutes. The percentage of time spent in each arm was analyzed using video tracking software (Ethovision XT).

The MWM test was performed using a white circular pool (120 cm in diameter), filled with warm (24°C–25°C), opacified water. The pretraining session (2 days) consisted of handling for 5 minutes and acclimation on a visible platform (10 cm in diameter) for 2 minutes, with 1 trial per day. During the 5-day training period of the hidden platform version of the MWM test, mice were allowed to find the submerged platform in 3 consecutive trials per day and were guided to the platform if they did not find the platform within 90 seconds. The starting position (opposite quadrant, right adjacent quadrant, and left adjacent quadrant) of each mouse was alternated between trials in a pseudo-random order. Each mouse was allowed to remain on the platform for 30 seconds, followed by a 30-second rest in its home cage. Mice were allowed to locate the hidden platform at a different starting point. The probe test was performed 24 hours after completion of the training trials, and mice were allowed to swim for 60 seconds in the absence of a hidden platform. The escape latency, swim distance, speed, and swim pattern were analyzed using video tracking software (Ethovision XT).

The visible platform version of the MWM test was performed using the same MWM pool but different mouse cohorts. After a 2-day pretraining session consisting of handling (5 minutes) and acclimation (2 minutes) on a visible platform once daily, the mice were trained to navigate to the visible platform for 5 days with 2 consecutive trials per day. The platform was raised 0.5 cm above the water surface, and its location was indicated by a high-contrast striped tube hung from the ceiling and placed 15 cm above the platform. Each mouse was allowed a maximum of 90 seconds to find the visible platform, and escape latency and swim speed were analyzed using video tracking software (Ethovision XT).

### Surgery and stereotaxic injection.

Mice (5−6 weeks old) were deeply anesthetized with a mixture of zoletil (50 mg/kg, i.p.) and xylazine (1 mg/kg, i.p.) and placed in a stereotaxic device. The skin was cut over the midline and craniotomies were performed bilaterally over the dorsal hippocampus (−1.9 anteroposterior, ±1.5 mediolateral, −1.5 dorsoventral from the bregma and dura). AAV vectors expressing Grin2b shRNA (5′-TgtaccaacaggtctcaccttaaacTTCAAGAGAgtttaaggtgagacctgttggtacTTTTTTC-3′) and scrambled shRNA (5′-TaccatcttgacataagcgacctcaTTCAAGAGAtgaggtcgcttatgtcaagatggtTTTTTTC-3′) were provided by Ronald Duman (Yale School of Medicine), and AAVs (AAV-DJ-shGrin2b, 1.41 × 10^13^ viral genome [vg]/mL; AAV-DJ–nontargeting scrambled sequence, 2.96 × 10^13^ vg/mL) were produced and titrated by the Stanford University Gene Vector and Virus Core.

Purified AAV (0.5 μL/side) was injected using a Hamilton syringe at a rate of 100 nL/min. Following completion of the injection, the needle (33G) was maintained in place for an additional 10 minutes to allow diffusion of the injection medium before being carefully retracted to prevent backflow. Experiments were performed 3−4 weeks after viral injections.

### Fiber photometry.

Under deep anesthesia with a mixture of zoletil and xylazine, AAV5/Syn-Flex-GCaMP6s-WPRE-SV40 (2.9 × 10^13^ vg/mL; Addgene; catalog 100845) was diluted 1:10 and unilaterally injected (0.5 μL) into the mPFC (+1.8 anteroposterior, ±0.5 mediolateral, −2.5 dorsoventral) of mice at 5−6 weeks age, after which a cannula (400 μm diameter, 0.39 NA; catalog CFM14U-20; Thorlabs) for fiber photometric recording was implanted above the injection site. The implants were secured using dental cement and metal screws anchored to the skull of the contralateral hemisphere. The mice were then allowed to recover for 3 weeks after surgery.

Ratiometric fiber photometry in the mPFC was conducted using an RZ5P processor running Synapse software (Tucker-Davis Technologies). A 405 nm LED (Doric Lenses) and 470 nm LED (Doric Lenses) were modulated at 211 and 531 Hz to detect Ca^2+^-independent isosbestic signals and Ca^2+^-dependent signals, respectively. Light from the LEDs and GCaMP6s fluorescence was passed through a minicube (catalog iFMC6; Doric Lenses) and the emitted light was detected using a fluorescence detector (Doric Lenses). Light power (10–30 μW) was measured at the tip of the fiber and adjusted using a light source device (catalog LDFLS4; Doric Lenses). Fluorescence signals (1 kHz) were low-pass filtered with a frequency cutoff of 10 Hz and demodulated to 381 Hz using a MATLAB script. The time course of photobleaching was estimated by double-exponential fitting of the fluorescence signals for the entire period, and photobleaching was corrected using a custom macro written in Igor Pro (WaveMetrics). The ΔF/F was calculated by dividing the change in the F signal by the baseline signal level.

### Immunohistochemistry and Western blotting.

For immunohistochemistry, mice (10−13 weeks old) were deeply anesthetized with diethyl ether and transcardially perfused with heparinized (10 U/mL) PBS, followed by PBS-buffered 4% (w/v) paraformaldehyde (PFA). Brains were removed, post-fixed in 4% PFA for 48 hours at 4°C, and cut into 100 μm coronal sections using a vibratome (catalog VT1200S; Leica). Sections were post-fixed (1 hour), permeabilized with 0.3% (v/v) Triton X-100 in PBS, and incubated in blocking buffer (5% normal goat serum, 5% horse serum, 5% donkey serum, and 0.5% BSA in PBS) for 2 hours. Sections were successively incubated with primary (anti-mCherry; [Abcam; catalog ab167453] and anti-GFP [Synaptic Systems; catalog 132 004] overnight at 4°C) and fluorescence (Cy3, Alexa fluor 647 or FITC: Jackson ImmunoResearch Laboratories) conjugated secondary (3 hours at room temperature) antibodies. Between each step, the sections were rinsed 3 times for 10 minutes in PBS. Images were acquired using an A1 confocal laser scanning microscope and processed using the NIS Viewer (Nikon). Wide-field images of entire brain sections were acquired using an LSM 980 confocal microscope and Zeiss Application Suite ZEN (Zeiss).

Western blotting was performed using protein samples prepared from 6- to 9-week-old mice. Hippocampi or whole brains were homogenized in a lysis buffer (50 mM HEPES, 100 mM NaCl, 5 mM EGTA, 5 mM EDTA, and 1% Triton X-100, pH 7.4) containing a phosphatase inhibitor cocktail (GenDEPOT; catalog P3200) and protease inhibitor cocktail (Sigma-Aldrich; catalog P8340). Hippocampal CA1 sections were sonicated using a probe sonicator on ice in lysis buffer. To prepare subcellular hippocampal fractions, homogenates of the hippocampus were centrifuged at 1,200*g* for 10 minutes to remove nuclei and other large debris (P1). The supernatant (S1) was centrifuged at 15,000*g* for 20 minutes to obtain the crude synaptosomal fraction (P2). All the steps were performed using a synaptic protein extraction reagent (Syn-PER; Thermo Fisher Scientific).

The protein concentration in each sample was determined using the Bradford Protein Assay kit (BIO-RAD; catalog 5000201), and proteins (10−15 μg) were separated using SDS-PAGE. Afterward, proteins were transferred from polyacrylamide gels (5% acrylamide, 0.05% bisacrylamide, pH 6.8 for the stacking gel, and 7.5%−10% acrylamide, 0.075%−0.1% bisacrylamide, pH 8.8 for the separating gel) to nitrocellulose membranes. The membranes were successively incubated with primary and horseradish peroxidase–conjugated secondary antibodies (Jackson ImmunoResearch Laboratories). Signals were detected using enhanced chemiluminescence (Cytiva). Quantification of bands was performed by measuring the integrated intensity of each band using MetaMorph software (Molecular Devices) and normalizing to α-tubulin or PSD-95 as loading controls. The antibodies against GluA1 and GluA2 have been described (68). The following primary antibodies were purchased from commercial suppliers: anti-GluN1 (catalog 556308), anti-GluN2A (catalog 612286), and anti-GluN2B (catalog 610416) from BD Biosciences; anti-PSD95 (catalog MA1-045) from Invitrogen; anti-GluN1 (catalog 5704S), anti-eEF2 (catalog 2332S), anti–p-eEF2 (catalog 2331S), and anti–GluA1-p-S845 (catalog 8084) from Cell Signaling Technology; anti–GluA2-p-S880 (catalog ab52180) from Abcam; anti–GABA_A_R-α5 (catalog 224 503) from Synaptic Systems; and anti–α-tubulin (catalog T5168) from Sigma-Aldrich.

### Statistics.

Statistical analyses were performed using Igor Pro (WaveMetrics) and SPSS software (IBM). The normality of the collected data was determined using the Shapiro-Wilk test. The Mann-Whitney *U* test, Wilcoxon signed-rank test, or Kruskal-Wallis test was used to compare non-normally distributed samples. Normally distributed samples were compared using a 2-tailed Student’s *t* test. For multiple groups, a 1-way or 2-way ANOVA, followed by Tukey’s honest significant difference post hoc test, was used to compare the samples. *P* values less than 0.05 were considered significant. All bar graphs in the figures show the mean ± SEM. The numbers of cells, sections, and animals used for each experiment and statistical analyses are provided in the Supporting Data Values file.

### Study approval.

All animal maintenance and experiment protocols were approved by the Institutional Animal Care and Use Committee of Seoul National University.

### Data availability.

Values for all data points in graphs are reported in the Supporting Data Values file. The data that support the findings of this study are available from the corresponding author upon reasonable request.

## Author contributions

WSS, YSB, and MHK conceived the project and designed the experiments. WSS, YSK, YSB, SHY, and JML performed the experiments and analyzed the data. WSS and MHK wrote the manuscript with input from all other authors.

## Supplementary Material

Supplemental data

Unedited blot and gel images

Supporting data values

## Version 1 05/08/2025

In-Press Preview

## Version 2 07/01/2025

Electronic publication
