# Supplementary material for: GluN2B suppression restores phenylalanine-induced neuroplasticity and cognition impairments in a mouse model of phenylketonuria
Source: J Clin Invest. 2025 May 8;135(13):e184299. doi: 10.1172/JCI184299 (PMC12208540; doi:10.1172/JCI184299)
Supplement: Supplemental data [file jci-135-184299-s216.pdf]

Supplemental Material

GluN2B suppression restores phenylalanine-induced neuroplasticity and cognition impairments in a mouse model of phenylketonuria

Woo Seok Song, Young Sook Kim, Young-Soo Bae, Sang Ho Yoon, Jae Min Lim, and Myoung-Hwan Kim

Supplemental Data: Supplemental Figure 1–9

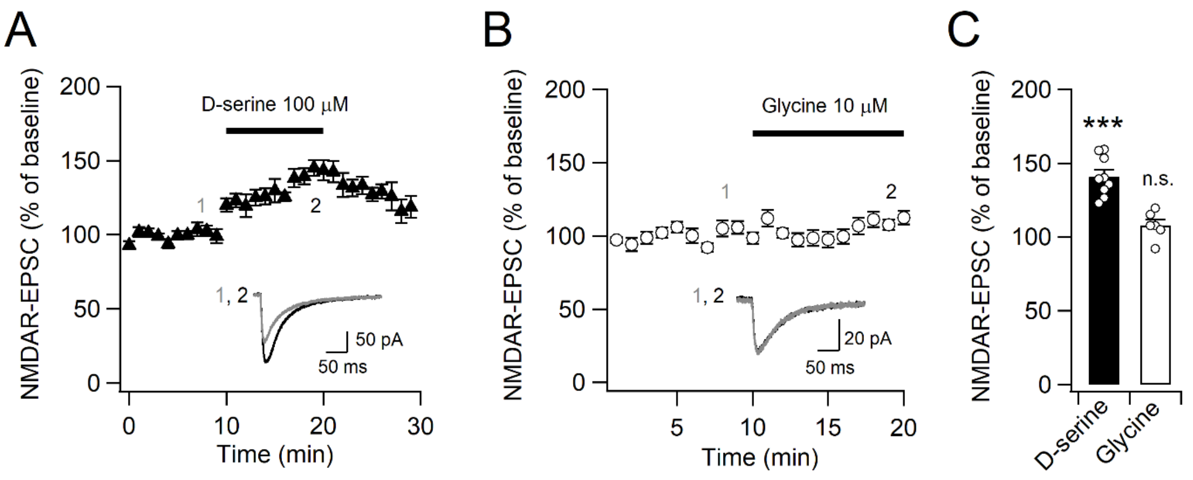

Supplemental Figure 1. NMDAR-EPSCs at SC-CA1 synapse were increased by D-serine. (**A**) Application of 100 µM D-serine increases the peak amplitude of NMDAR-EPSCs. Inset: sample traces of NMDAR-EPSCs measured at indicated time points (1, 2). (**B**) Addition of glycine to ACSF did not affect NMDAR-EPSCs. Time course of the peak amplitude of NMDAR-EPSCs before and during bath application of 10 µM glycine. The inset shows representative traces of NMDAR-EPSCs obtained before and during perfusion with 10 µM glycine. (**C**) Mean amplitudes of NMDAR-EPSCs in the presence of D-serine or glycine in the ACSF were compared with those obtained during baseline recording. Statistical analysis was performed using Student's *t*-test. ***$P < 0.001$, and n.s., not significant ($P \geq 0.05$).

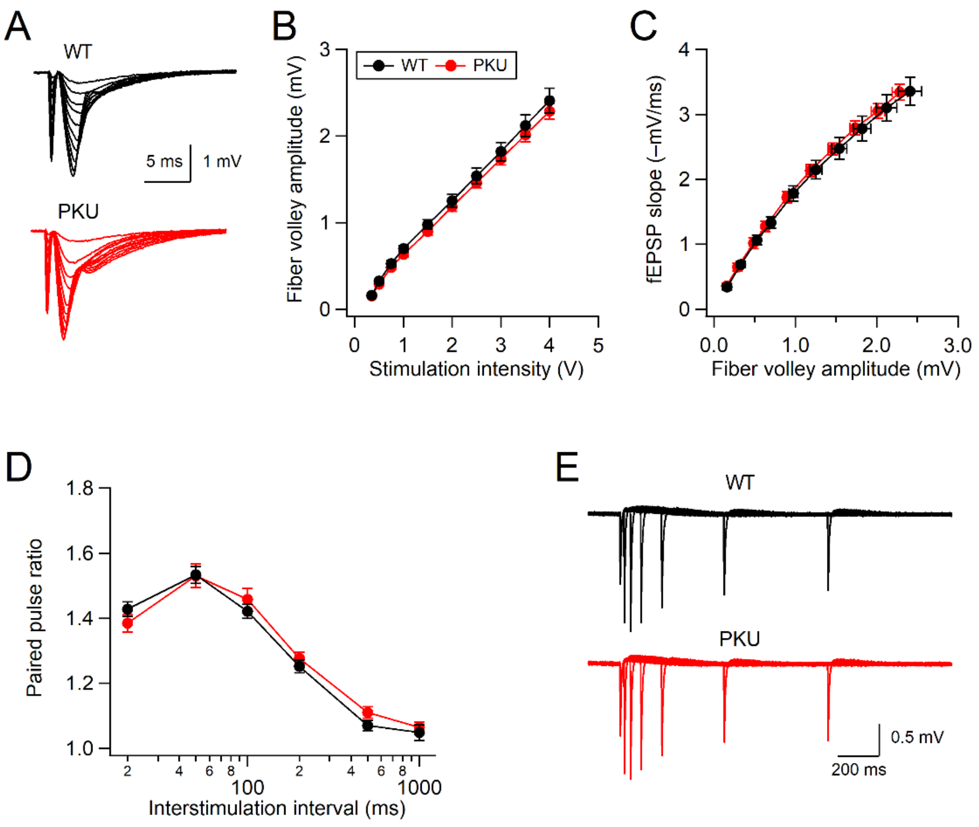

Supplemental Figure 2. Basal synaptic transmission at SC-CA1 synapse is normal in *Pah*^{Enu2} mice. (**A**) fEPSPs were evoked in WT and *Pah*^{Enu2} hippocampal slices at different stimulation intensities. (**B**) The amplitude of the fiber volley is plotted against stimulation intensity. (**C**) The synaptic input-output ratios determined by plotting fEPSP slopes against fiber volley amplitudes were not different between WT and *Pah*^{Enu2} mice. (**D**) Paired-pulse ratios of fEPSP slopes from WT and *Pah*^{Enu2} slices are plotted as a function of the interstimulation interval. (**E**) Sample traces of fEPSPs evoked by two consecutive stimuli at different interstimulus intervals.

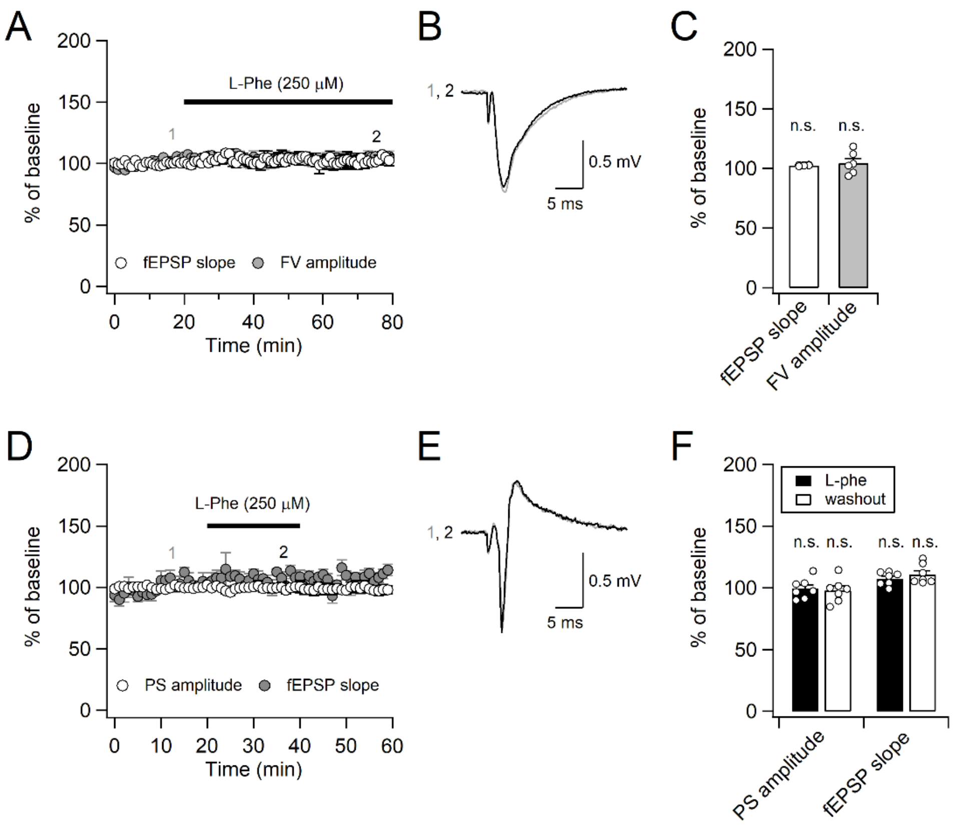

Supplemental Figure 3. L-Phe did not affect AMPAR-mediated synaptic transmission and population spikes (PS) in CA1 neurons. (**A**) The slopes of fEPSPs and fiber volley (FV) amplitudes measured at the SC-CA1 synapse plotted against the recording time. (**B**) Sample fEPSP traces obtained before and during L-Phe perfusion. (**C**) The slopes of the fEPSPs and FV amplitudes were not affected by L-Phe perfusion. (**D**) The recording electrode was placed in the CA1 stratum pyramidale, and fEPSP and PS were induced by stimulating SC axons. The slopes of the fEPSPs and PS amplitudes were normalized to those obtained at baseline and plotted as a function of time. (**E**) Sample fEPSP and PS traces obtained before and during L-Phe perfusion. (**F**) The slopes of fEPSPs and the amplitudes of PSs during and after L-Phe perfusion were summarized. Statistical analysis was performed using the Student's *t*-test (**C**) or one-way ANOVA with post-hoc Tukey's test (**F**). n.s., not significant ($P \geq 0.05$).

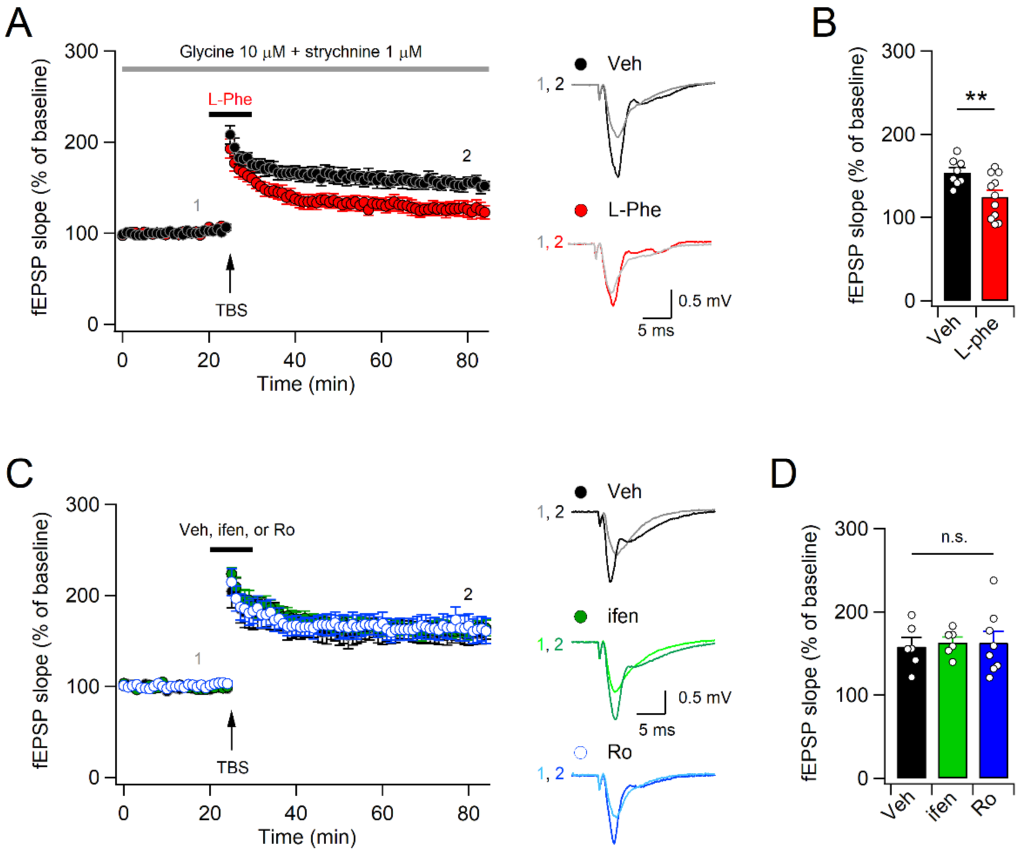

Supplemental Figure 4. The effects of L-Phe and GluN2B inhibitors on the LTP at hippocampal SC-CA synapses. (A, B) L-Phe attenuates NMDAR-dependent LTP in the presence of glycine. (**A**) The slopes of fEPSPs measured in the presence of added (10 μM) glycine and the glycine receptor antagonist strychnine (1 μM) were plotted as a function of time. Right: sample traces obtained during baseline and the last 10 min of recording. (**B**) The magnitude of LTP during the last 10 min of recording was significantly reduced by brief L-Phe perfusion during the peri-TBS period. (**C**, **D**) GluN2B inhibition did not affect TBS-induced LTP at the SC-CA1 synapse. (**C**) Time course of fEPSP slopes (left) and sample traces (right) of fEPSPs obtained during baseline and the last 10 min of recordings. Ro (2 μM) and ifen (6 μM) were perfused for 10 min starting 5 min before TBS. (**D**) Magnitudes of LTP measured under each condition. Statistical analysis was performed using the Student's *t*-test (**B**) or one-way ANOVA with post-hoc Tukey's test (**D**). $^{**}P < 0.01$, and n.s., not significant ($P \geq 0.05$).

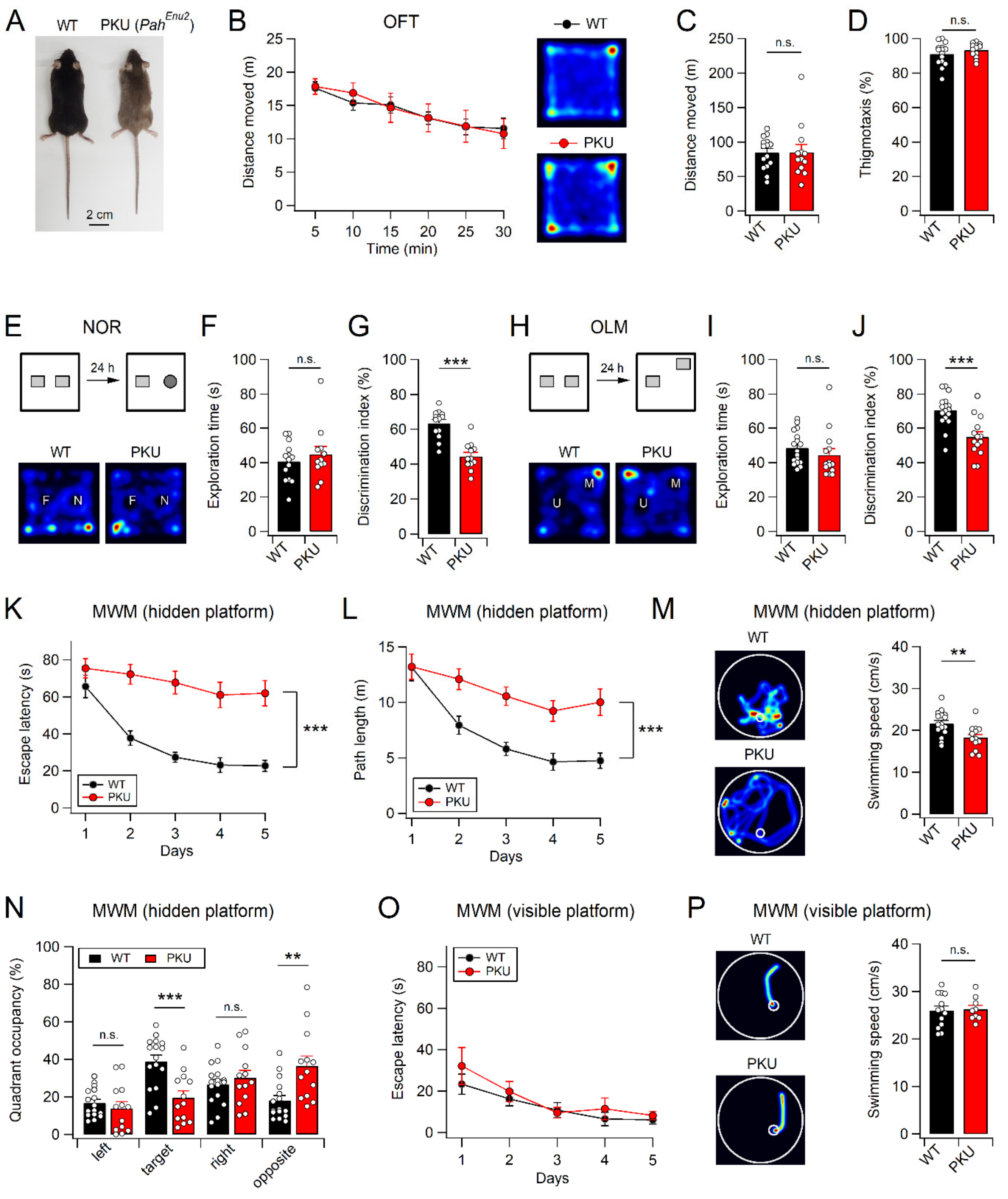

Supplemental Figure 5. ***Pah**^{Enu2}* **mice display impaired hippocampus-dependent learning and memory.** (**A**) Representative images of 8-week-old male WT and *Pah*^{Enu2} mice. (**B–D**) Normal open field activity in *Pah*^{Enu2} mice. (**B**) The activity of mice in the open-field box was plotted in 5 min bins for 30 min. Right, the representative activity path of mice during the first 10 min of the OFT test is shown. The distance moved of mice during the entire period (**C**) and

the percentage of time spent in the outer region of the open field box during the first 15 min (**D**) are summarized. (**E–J**) *Pah*^{Enu2} mice display impaired NOR (**E–G**) and OLM (**H–J**). (**E**) Experimental design for the NOR tests and representative activity paths of mice during the NOR test session. F, familiar object; N, novel object. Time spent exploring familiar and novel objects (**F**) and discrimination index (**G**) for NOR during the test session. (**H–J**) Representative activity path (**H**), time spent exploring the two objects (**I**), and relative preference for the moved object (**J**) during the OLM test session. (**H**) M, moved object; U, unmoved object. (**K–N**) Impaired MWM performance in *Pah*^{Enu2} mice. Escape latency (**K**) and swim distance (**L**) to find the hidden platform during the training period of MWM test. (**M**, **N**) Representative swim path (**M**, left), swimming speed (**M**, right), and quadrant occupancy (**N**) of WT and *Pah*^{Enu2} mice during the probe trials of the hidden platform MWM test. (**O**, **P**) Normal performance of *Pah*^{Enu2} mice during the visible platform sessions of MWM test. (**O**) Escape latency to reach the visible platform during the 5-day training period of MWM test. (**P**) Representative swim paths (left) and swimming speed (right) of WT and *Pah*^{Enu2} mice during the second trial on the last day of the visible platform MWM test. Statistical analysis was performed using Mann–Whitney U test (**C**, **F**, **I**, and **N**), Student's *t*-test (**D**, **G**, **J**, **M**, **N**, and **P**), or two-way ANOVA with post-hoc Tukey's test (**K**, **L**, and **O**). $^{**}P < 0.01$, $^{***}P < 0.001$, and n.s., not significant ($P \geq 0.05$).

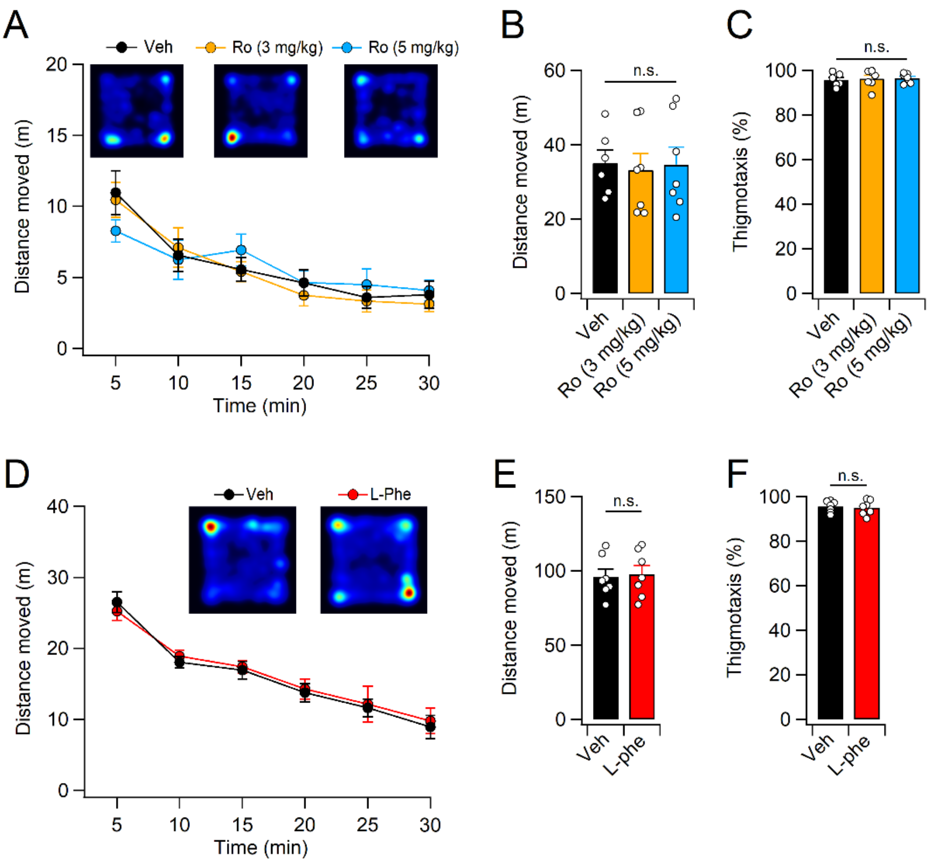

Supplemental Figure 6. Low concentrations of Ro and L-Phe did not affect open field activity of mice. (**A**) The activity of mice in the open-field box was monitored for 30 min starting 1 h after Ro administration (3 or 5 mg/kg, i.p.). (**B**) Mice treated with 3 or 5 mg/kg Ro moved similar distances to those that received the vehicle. (**C**) Vehicle- and Ro-treated mice spent similar amounts of time in the outer region of the open field box. (**D**) The OFT was performed 30 min after L-Phe administration. (**E, F**) L-Phe challenge (1 mg/g, i.p.) did not affect the distance moved (**E**) or thigmotactic behavior (**F**) of mice during the OFT. Statistical analysis was performed using Student's *t*-test (**E** and **F**) or one-way ANOVA (**B** and **C**) with post hoc Tukey's test. n.s., not significant ($P \geq 0.05$).

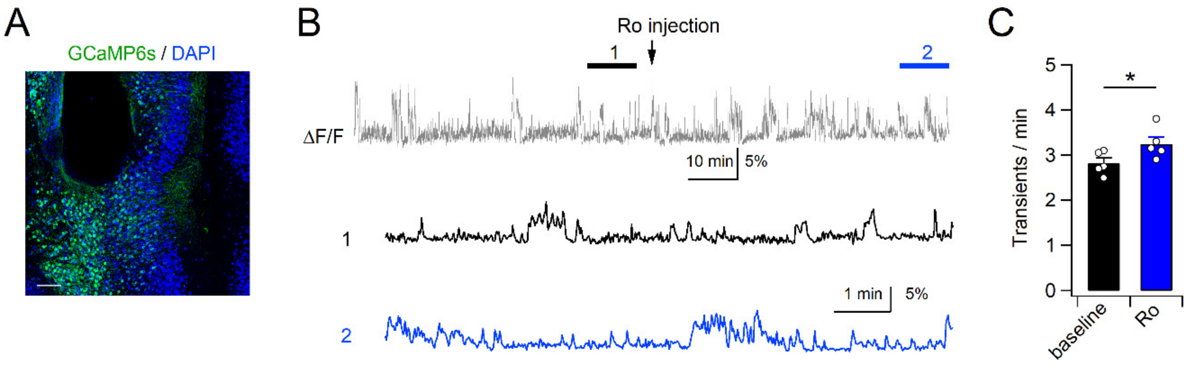

Supplemental Figure 7. Ro administration increases neural activity in the mPFC of *Pah*^{Enu2} mice. (**A**) Immunofluorescence image showing GCaMP6s expression and cannula placement in the *Camk2a-Cre*;*Pah*^{Enu2} mPFC. Calibration, 200 μm. (**B**) Neural activity was monitored by fiber photometry. After 60 min of baseline recording, *Camk2a-Cre*;*Pah*^{Enu2} mice were administered Ro (3 mg/kg, i.p.). (Bottom) Fluorescence signals obtained during the indicated periods (1 and 2) are shown on an expanded time scale. (**C**) The frequency of Ca^{2+} transients before and after Ro administration. *P < 0.05, Student's *t*-test.

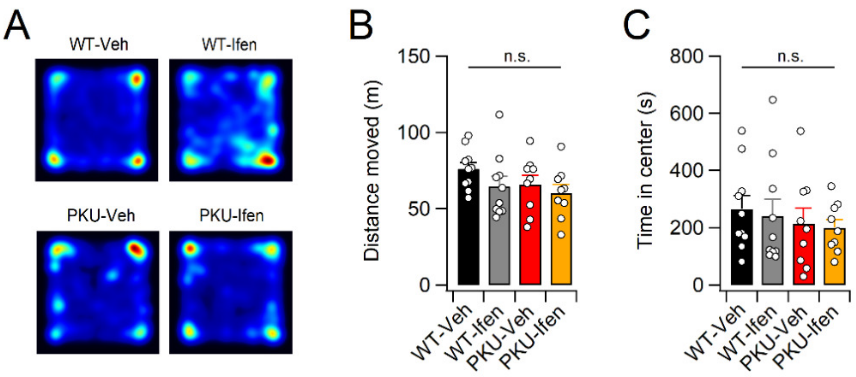

Supplemental Figure 8. Ifen did not affect the open field activity of WT and *Pah*^{Enu2} mice. (**A**) Sample activity paths of mice during the OFT. Mice were treated with ifen (5 mg/kg, i.p.) or vehicle 30 min before the OFT. (**B**, **C**) Pretreatment with ifen had no effect on the distance moved (**B**) or time spent (**C**) in the central area during the OFT. n.s., not significant ($P \geq 0.05$), two-way ANOVA with post-hoc Tukey's test.

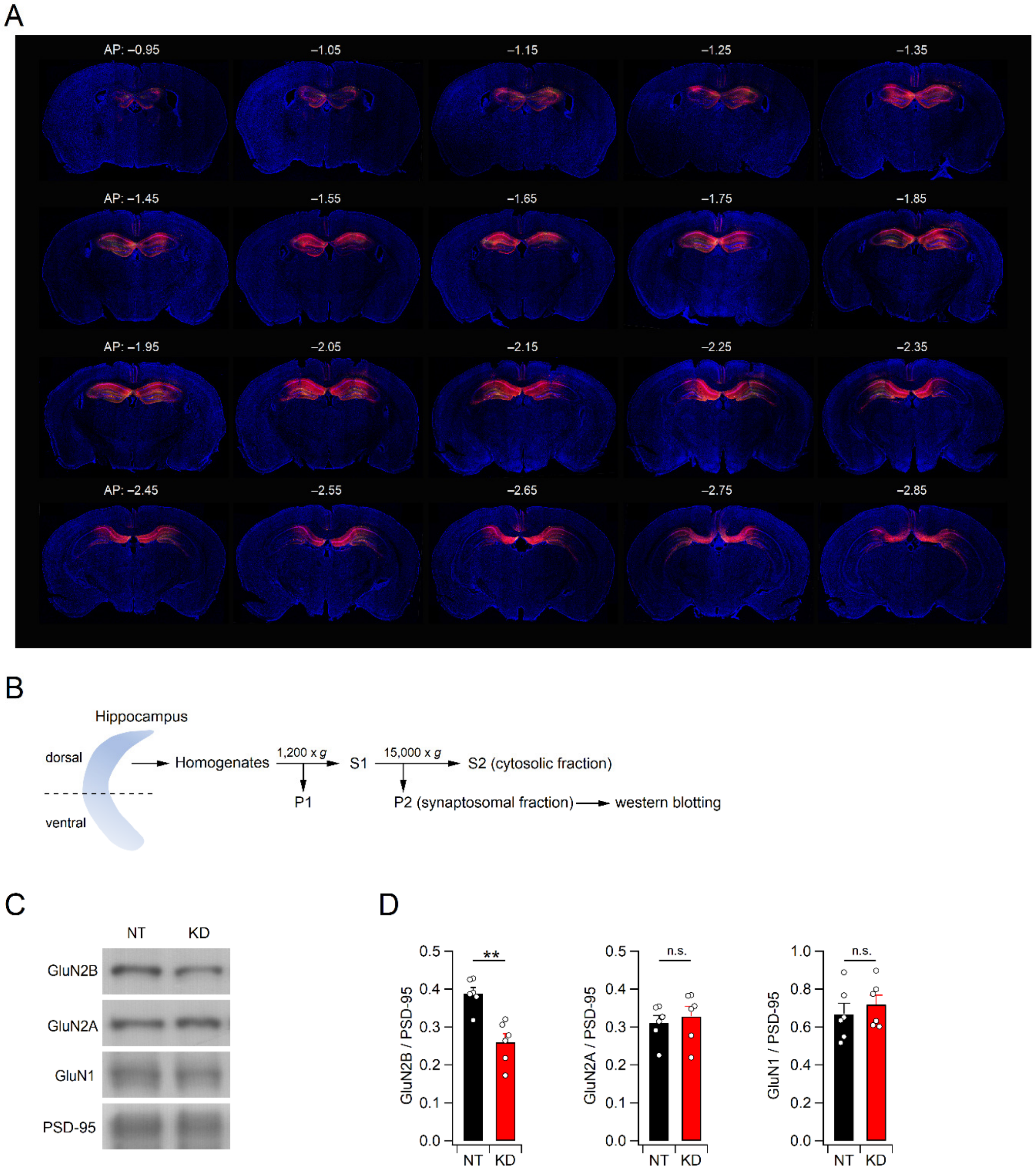

Supplemental Figure 9. Histochemical analysis of mCherry distribution and western blot analyses of GluN2B knockdown efficiency. (**A**) A series of fluorescence images showing the distribution of shGrin2b-expressing cells in the hippocampi of *Camk2a-Cre*;*Pah*^{Enu2} mice. Numbers represent the coordinates of the anterior-posterior (AP) position from bregma (mm). DAPI (blue) was used to identify the brain regions and structures. (**B–D**) Reduced GluN2B

expression in the dorsal hippocampus of *Camk2a-Cre* mice expressing shGrin2b. (**B**) Diagram showing the preparation of the synaptosomal fraction from the dorsal hippocampus. (**C**) The expression levels of GluN1, GluN2A, and GluN2B were analyzed by western blotting. PSD-95 was used as a loading control for normalization. NT and KD represent shNT and shGrin2b expression, respectively. (**D**) *Camk2a-Cre* mice infected with AAV-shGrin2b exhibited reduced expression of GluN2B but not GluN1 or GluN2A in the synaptosomal fraction of the dorsal hippocampus compared to those infected with AAV-shNT. **$P < 0.01$ and n.s., not significant ($P \geq 0.05$) by Student's *t*-test.
