# Supplementary material for: GluN2B suppression restores phenylalanine-induced neuroplasticity and cognition impairments in a mouse model of phenylketonuria
Source: J Clin Invest. 2025 May 8;135(13):e184299. doi: 10.1172/JCI184299 (PMC12208540; doi:10.1172/JCI184299)

Figure 2A (whole hippocampus)

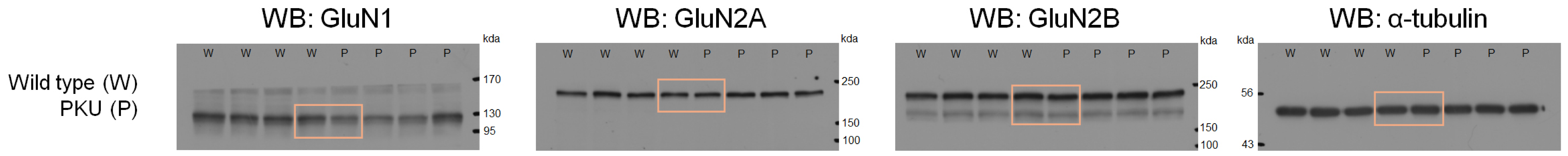


Figure 2C (synaptosomal fraction)

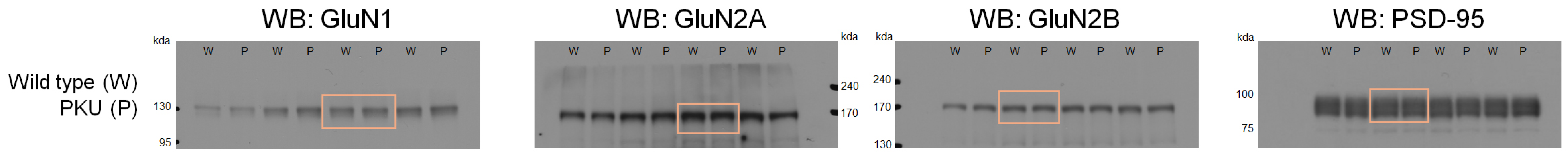


Figure 2J (hippocampus)

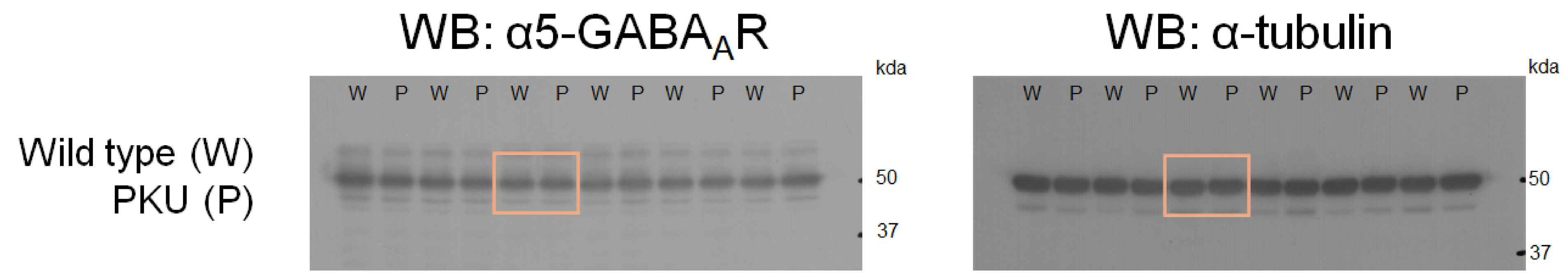


Figure 3G (hippocampal slices)

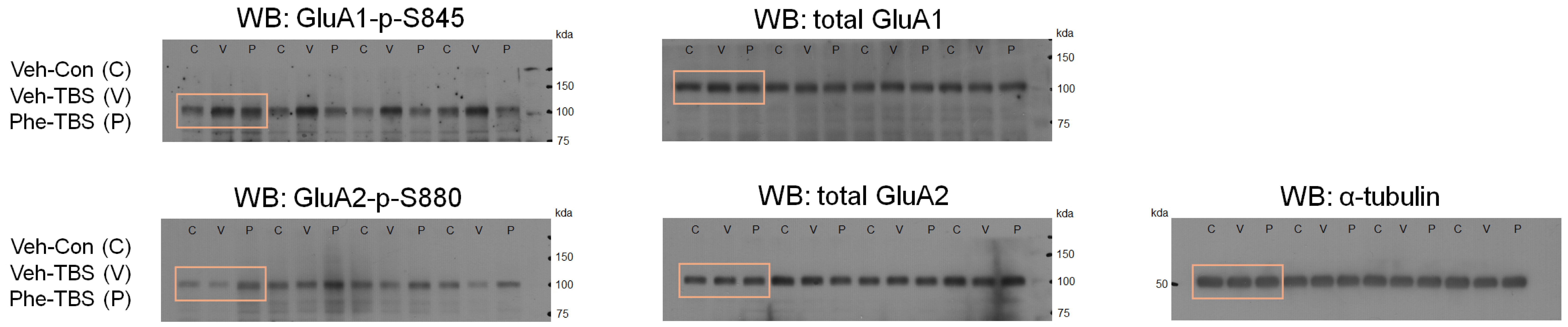


Figure 4C (whole brain)

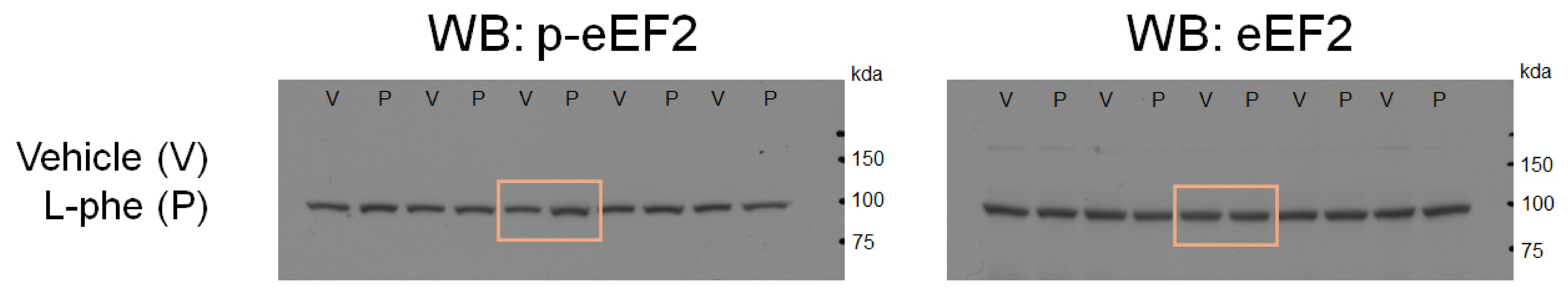


Figure 4C (hippocampus)

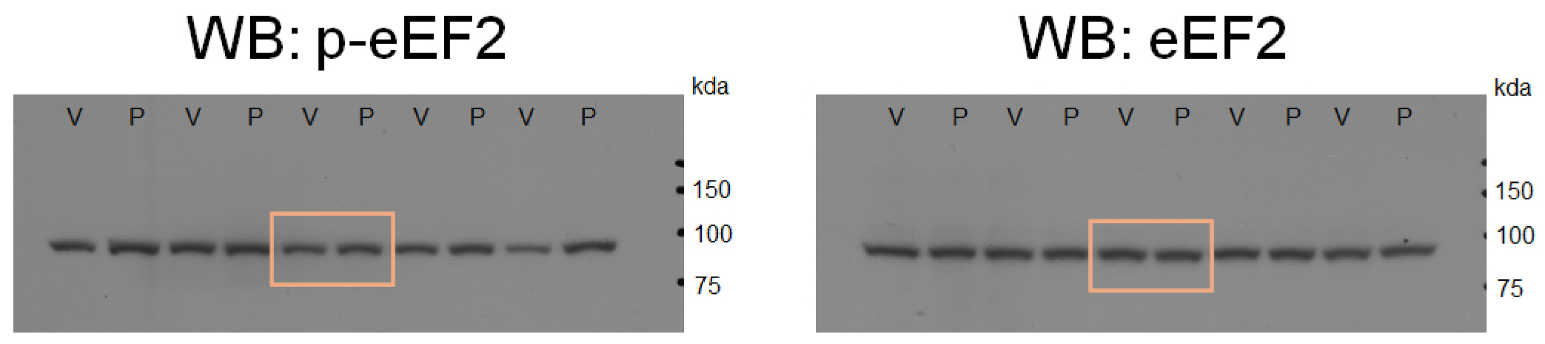


Figure 4D (hippocampus)

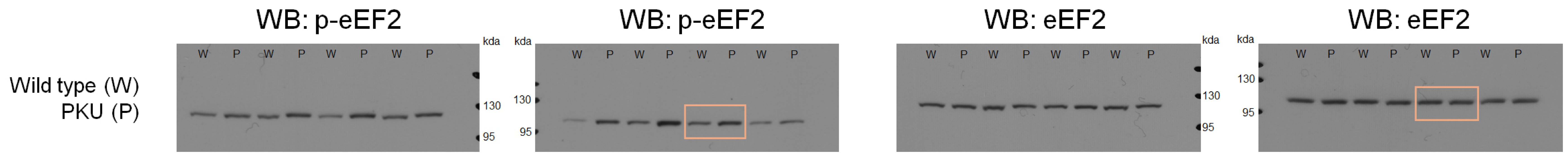


Figure 4H (whole brain)

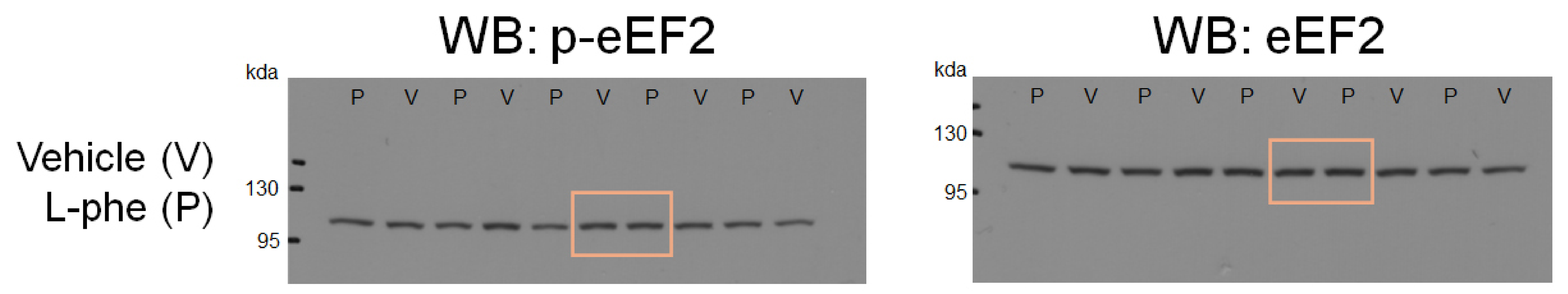


Figure 4H (hippocampus)

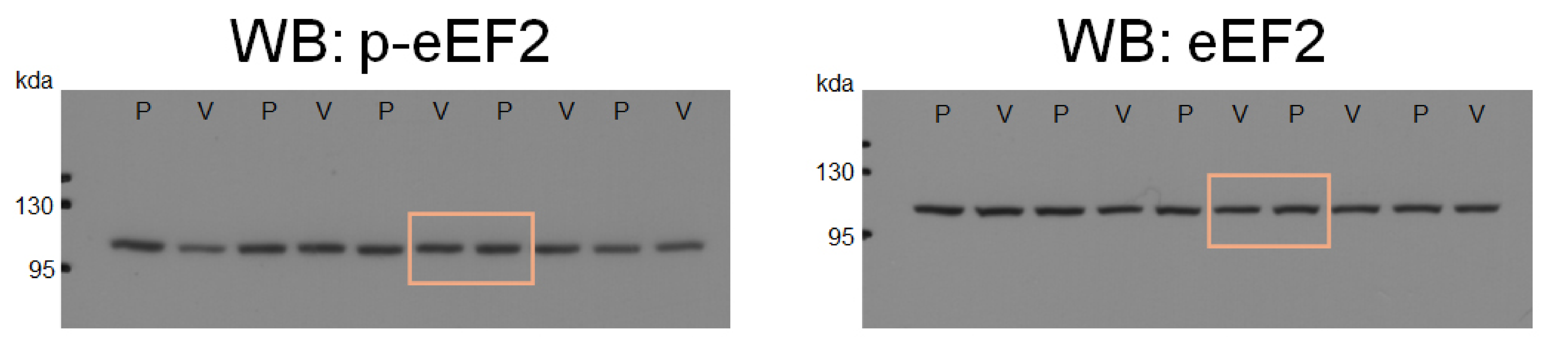


Figure S9C (dorsal hippocampus, synaptosomal fraction)

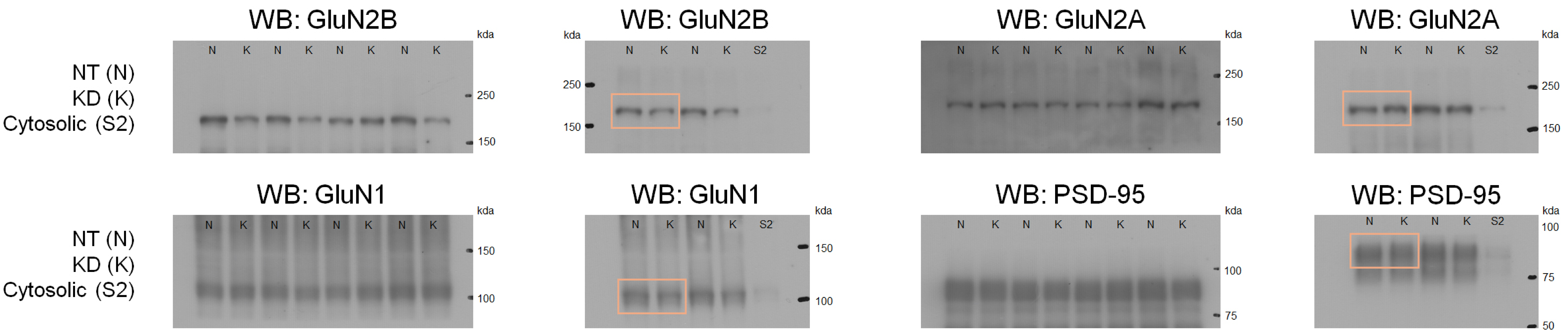

Supplement: Unedited blot and gel images [file jci-135-184299-s217.pdf]
